# TrkA Interacts with and Phosphorylates STAT3 to Enhance Gene Transcription and Promote Breast Cancer Stem Cells in Triple-Negative and HER2-Enriched Breast Cancers

**DOI:** 10.3390/cancers13102340

**Published:** 2021-05-12

**Authors:** Angelina T. Regua, Noah R. Aguayo, Sara Abu Jalboush, Daniel L. Doheny, Sara G. Manore, Dongqin Zhu, Grace L. Wong, Austin Arrigo, Calvin J. Wagner, Yang Yu, Alexandra Thomas, Michael D. Chan, Jimmy Ruiz, Guangxu Jin, Roy Strowd, Peiqing Sun, Jiayuh Lin, Hui-Wen Lo

**Affiliations:** 1 Department of Cancer Biology, Wake Forest University School of Medicine, Winston-Salem, NC 27101, USA; aregua@wakehealth.edu (A.T.R.); raguayo@highpoint.edu (N.R.A.); sabujal@wakehealth.edu (S.A.J.); ddoheny@wakehealth.edu (D.L.D.); smanore@wakehealth.edu (S.G.M.); dozhu@wakehealth.edu (D.Z.); glwong@wakehealth.edu (G.L.W.); aarrigo@wakehealth.edu (A.A.); cjwagner@wakehealth.edu (C.J.W.); yayu@wakehealth.edu (Y.Y.); gjin@wakehealth.edu (G.J.); psun@wakehealth.edu (P.S.); 2 Department of Hematology and Oncology, Wake Forest University School of Medicine, Winston-Salem, NC 27101, USA; althomas@wakehealth.edu (A.T.); jruiz@wakehealth.edu (J.R.); 3 Breast Cancer Center of Excellence, Wake Forest University School of Medicine, Winston-Salem, NC 27101, USA; 4 Wake Forest Baptist Comprehensive Cancer Center, Wake Forest University School of Medicine, Winston-Salem, NC 27101, USA; mchan@wakehealth.edu (M.D.C.); rstrowd@wakehealth.edu (R.S.); 5 Department of Radiation Oncology, Wake Forest University School of Medicine, Winston-Salem, NC 27101, USA; 6 Department of Neurology, Wake Forest University School of Medicine, Winston-Salem, NC 27101, USA; 7 Department of Biochemistry and Molecular Biology, University of Maryland School of Medicine, Baltimore, MD 21201, USA; jlin@som.umaryland.edu

**Keywords:** breast cancer, TrkA, STAT3, cancer stem cells, SOX2, MYC

## Abstract

**Simple Summary:**

Breast cancer is the leading cancer in American women. Due to the inherent aggressiveness of triple-negative and HER2-enriched breast cancers, it is imperative to identify novel molecular targets for therapeutic intervention. Due to their abnormal activities in metastatic breast cancers, JAK2–STAT3 and TrkA pathways have been individually implicated in aggressive breast tumors. However, their co-activation and signaling interactions have not been thoroughly investigated. Therefore, our study aimed to elucidate the extent of crosstalk between JAK2–STAT3 and TrkA signaling pathways and its impact on breast cancer. Our data revealed a novel interaction between TrkA and STAT3, and that this interaction results in STAT3 phosphorylation and activation by TrkA, leading to enhanced stemness gene expression and stem cell renewal. We further found that the co-activation of JAK2–STAT3 and TrkA pathways is correlated with shorter time to develop overall and organ-specific metastasis, suggesting that this signaling crosstalk underlies the aggressiveness of triple-negative and HER2-enriched breast cancers.

**Abstract:**

JAK2–STAT3 and TrkA signaling pathways have been separately implicated in aggressive breast cancers; however, whether they are co-activated or undergo functional interaction has not been thoroughly investigated. Herein we report, for the first time that STAT3 and TrkA are significantly co-overexpressed and co-activated in triple-negative breast cancer (TNBC) and HER2-enriched breast cancer, as shown by immunohistochemical staining and data mining. Through immunofluorescence staining–confocal microscopy and immunoprecipitation–Western blotting, we found that TrkA and STAT3 co-localize and physically interact in the cytoplasm, and the interaction is dependent on STAT3-Y705 phosphorylation. TrkA–STAT3 interaction leads to STAT3 phosphorylation at Y705 by TrkA in breast cancer cells and cell-free kinase assays, indicating that STAT3 is a novel substrate of TrkA. β-NGF-mediated TrkA activation induces TrkA–STAT3 interaction, STAT3 nuclear transport and transcriptional activity, and the expression of STAT3 target genes, *SOX2* and *MYC*. The co-activation of both pathways promotes breast cancer stem cells. Finally, we found that TNBC and HER2-enriched breast cancer with JAK2–STAT3 and TrkA co-activation are positively associated with poor overall metastasis-free and organ-specific metastasis-free survival. Collectively, our study uncovered that TrkA is a novel activating kinase of STAT3, and their co-activation enhances gene transcription and promotes breast cancer stem cells in TNBC and HER2-enriched breast cancer.

## 1. Introduction 

Breast cancer is the most commonly diagnosed cancer in American women and is the second-leading cause of cancer-related deaths among women [1]. Breast cancers can be categorized into five separate subtypes: luminal A (ER+/PR+), luminal B (ER+/PR+/HER2 ± with high Ki-67), normal breast-like, HER2-positive (HER2-enriched), and triple-negative (ER−/PR−/HER2−; TNBC). Triple-negative and HER2-enriched subtypes are diagnosed in up to 30% of breast cancer cases and are considered more aggressive than other subtypes. As opposed to surgical resection and adjuvant chemotherapy, most patients with triple-negative and HER2-positive breast cancer now undergo systemic neoadjuvant chemotherapy as a standard of care, showing high pathological response and positive long-term outcome [2,3]. However, regardless of treatment modality, many breast cancer patients often relapse and present with recurrent breast cancer that currently remains incurable. Consequently, patients with recurrent breast cancer experience high rates of morbidity and mortality [4]. As such, there is an urgent need to identify new molecular targets that drive breast cancer growth and progression and then develop therapeutic intervention against these targets.

Janus kinase 2 (JAK2) is a non-receptor tyrosine kinase that is frequently amplified or aberrantly active in triple-negative and HER2-enriched breast cancers [5,6]. JAK2 functions as a central signaling hub that links oncogenic receptor tyrosine kinase signaling and interleukin receptor activity to Signal Transducer and Activator of Transcription 3 (STAT3) transcription factor [7]. JAK2 phosphorylates STAT3 on its Y705 residue, resulting in the dimerization and nuclear translocation of STAT3 and activation of STAT3 transcriptional activity, leading to the expression of STAT3 target genes that promote cell proliferation, differentiation, survival, and migration [8,9,10]. We have previously reported that JAK2–STAT3 can upregulate the expression of TWIST, iNOS, COX-2, and STAT1 [11,12,13,14]. Since JAK2–STAT3 signaling is frequently upregulated in aggressive breast cancers and the upregulation is correlated to poor overall clinical outcome, the pathway has been regarded as an important target of cancer drug development. Of note, three orally active JAK2 inhibitors, ruxolitinib, baricitinib, and fedratinib, have been approved by the FDA for rheumatoid arthritis and myelofibrosis [15,16,17]; they are being evaluated for human cancers in numerous clinical trials.

Tropomyosin receptor tyrosine kinase A (TrkA) is a neurotrophic receptor encoded by the NTRK1 gene and is implicated in numerous cancer types, in part, due to its propensity for forming oncogenic NTRK fusion genes that drive the malignant progression of some cancer types [18]. However, wild-type TrkA has also been shown to function as a potent oncogenic driver when overexpressed in cells. The binding of its cognate ligand β-Nerve Growth Factor (β-NGF) results in TrkA receptor dimerization and activation, allowing for TrkA to phosphorylate its substrates and activate pathways, such as RAS/MAPK and PI3 kinase, resulting in increased cell proliferation and survival [19]. Similar to the JAK2–STAT3 signaling, the TrkA pathway is implicated in breast cancer progression. The overexpression of TrkA confers increased cell proliferation, migration, and metastatic capacity of breast cancer cells along with concomitant reduction in apoptosis [20,21,22,23]. TrkA-overexpressing breast tumors were found to grow significantly faster in vivo with increased development of lung, liver, and brain metastases, suggesting that TrkA activation contributes to breast cancer metastasis [20,21,24]. Accordingly, TrkA, as well as other members of the TRK protein family, have become therapeutic targets for inhibitor design [18]. Two orally active TrkA inhibitors, Larotrectinib and Entrectinib, have been approved by the FDA for treating solid tumors bearing NTRK fusion genes [25,26]. 

Although previous reports show that the activation of TrkA by its cognate ligand, β-NGF, promotes the phosphorylation of STAT3 on its Y705 and S727 residues and promotes STAT3 transcriptional activity, the co-activation of JAK2–STAT3 and TrkA signaling pathways, and the mechanisms underlying their crosstalk, have never been investigated in any cell or tumor type [27,28]. In this study, we report for the first time that signaling crosstalk between the JAK2–STAT3 and TrkA pathways is mediated through TrkA–STAT3 protein–protein interactions and TrkA-mediated phosphorylation of STAT3 Y705, leading to STAT3 activation and increased expression of STAT3 target genes. Immunohistochemical (IHC) analyses of human breast cancer samples reveal a high expression of p-STAT3 (Y705) and p-TrkA (Y490) in the tissue, supporting the co-activation of JAK2–STAT3 and TrkA pathways. Survival analyses of publicly available datasets reveal dismal survival and metastasis-free survival in patients with co-activation of JAK2–STAT3 and TrkA. Finally, we show that TrkA interacts directly with and phosphorylates STAT3 on Y705 to promote oncogenic gene transcription, implicating a novel mechanism to activate STAT3-mediated breast cancer metastasis in TrkA-overexpressing tumors. 

## 2. Results

### 2.1. JAK2–STAT3 and TrkA Pathways Are Significantly Co-Activated in Triple-Negative and HER2-Enriched Breast Cancers

**Figure 1:**
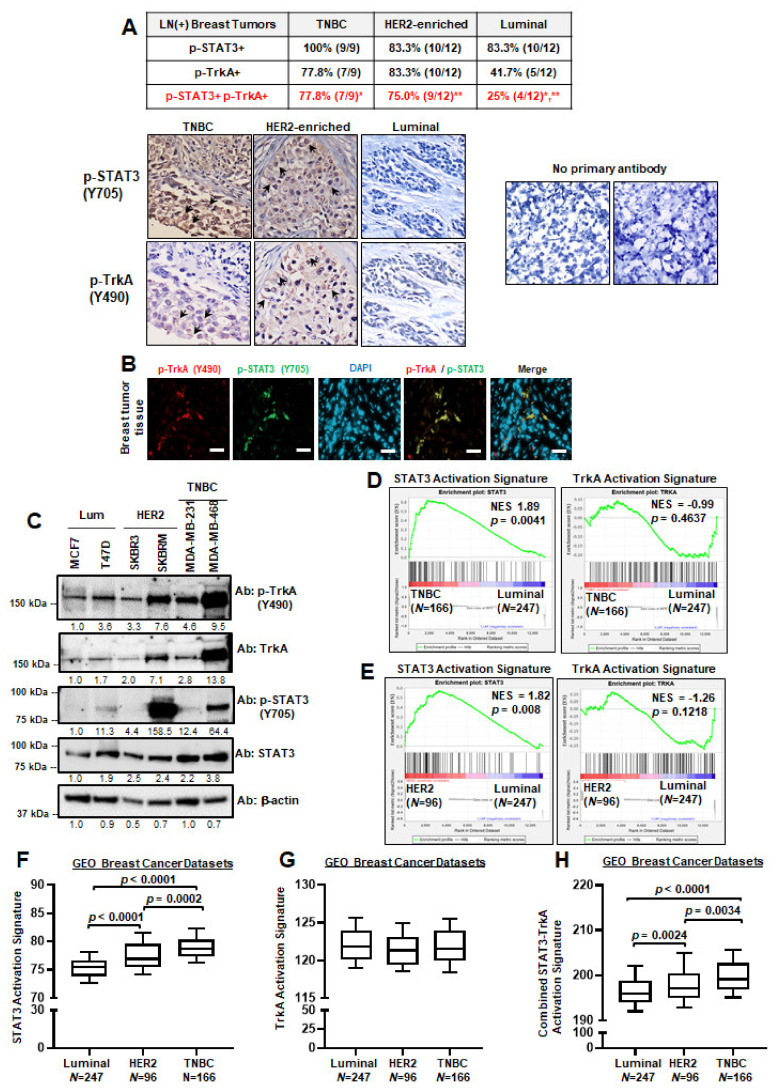
STAT3 and TrkA signaling pathways are significantly co-activated in triple-negative and HER2-enriched breast cancers. (**A**) IHC staining of 33 node-positive breast cancer tumors showed strong positive staining for both activated STAT3 (p-Y705) and activated TrkA (p-Y490) in both TNBC and HER2-enriched samples. Representative IHC images from three different subtypes of tumors are shown at the bottom. Arrows indicate positive staining for p-TrkA (Y490) at the cell membrane and nuclear staining for p-STAT3 (Y705). In a negative control experiment for IHC (no primary antibody, secondary antibody only), a mammary fat pad xenograft of BT474-TtzmR, a breast cancer cell line that is resistant to trastuzumab, was used. Magnification, 20×. (**B**) Immunofluorescent staining of patient breast tumor tissue for p-TrkA (Y490) and p-STAT3 (Y705). Scale bars, 50 μm. (**C**) Western blot panel of breast cancer cell lines to determine TrkA and STAT3 activation as indicated by p-TrkA and p-STAT3, respectively. (**D**,**E**) GSEA analysis of GEO breast cancer datasets (GSE 2034, 2603, 5327, 12,276) comparing STAT3 and/or TrkA activation signatures in TNBC versus luminal breast cancers (**D**) and HER2-enriched versus luminal tumors (**E**). (**F**–**H**) Activation scores for STAT3 (**F**), TrkA (**G**), and STAT3-TrkA (**H**) pathways in three major subtypes of breast cancer. GEO datasets were used. Original Western blots can be found in Supplementary Figure S3.

To determine the extent to which JAK2–STAT3 and TrkA signaling pathways are co-activated in breast tumor samples, we performed IHC staining on 33 node-positive breast carcinomas across three major subtypes (triple-negative, HER2-enriched, and luminal subtypes) to detect phosphorylated TrkA (Y490) and STAT3 (Y705) proteins. Results showed that node-positive triple-negative breast cancer (TNBC) and HER2-positive breast tumors highly co-express p-TrkA and p-STAT3Of the nine TNBC samples, we found that 100% of them have high p-STAT3 and 77.8% of these samples concurrently express high p-TrkA. Similarly, 83% of HER2-positive breast tumors were highly stained for both p-TrkA and p-STAT3. Although over 80% of node-positive luminal samples showed high p-STAT3 levels, only 41% of these samples also highly expressed p-TrkA. These results indicated that concurrently activated JAK2–STAT3 and TrkA signaling is preferentially found in TNBC and HER2-positive breast cancer. We found a 61% (20/33) positivity rate for p-TrkA and p-STAT3 co-overexpression after examining each individual sample. Representative IHC images are shown in Figure 1A-bottom. As expected, p-TrkA was primarily detected on cell membrane (arrows), while p-STAT3 was found primarily within the nucleus (arrows). Since STAT3 phosphorylation by receptor tyrosine kinases and non-receptor tyrosine kinases occur in the cytoplasm, diffuse cytoplasmic staining was also observed. In a negative control experiment for IHC (no primary antibody, secondary antibody only), we did not observe any positive staining from a mammary fat pad xenograft of BT474-TtzmR, which is a breast cancer cell line that is resistant to trastuzumab. Then, we confirmed our IHC findings using immunofluorescent staining of a primary patient breast tumor and found that cells with p-TrkA were also positive for p-STAT3 (Figure 1B), supporting our hypothesis that these two proteins are co-expressed and co-localized in breast tumor tissues. Furthermore, we expanded our investigation of TrkA and STAT3 co-activation by performing a Western blot analysis using breast cancer cell lines of varying subtypes (Figure 1C). Taken together, our findings suggest that the co-activation of JAK2–STAT3 and TrkA pathways may preferentially occur in TNBC and HER2-enriched breast cancers.

To validate the above-mentioned results, we analyzed publicly available breast cancer databases to determine the extent to which target genes downstream of the JAK2–STAT3 and TrkA pathways are enriched in triple-negative and HER2-enriched breast cancers. To this end, we applied Gene Set Enrichment Analyses (GSEA) using a STAT3 activation signature, TrkA activation signature, or a combination of STAT3 and TrkA signatures to examine 1533 breast cancer patient samples from TCGA and GEO databases [29,30,31,32,33,34,35]. Here, we observed that TNBC and HER2-enriched breast cancer showed significantly higher enrichment of the STAT3 activation signature, but not the TrkA activation signature, when compared to luminal samples (Figure 1D,E). Importantly, the combined STAT3 and TrkA activation signature was significantly enriched in triple-negative and HER2-enriched breast cancers in comparison to luminal subtypes (Supplementary Figure S1A,B). Further analysis of the GEO datasets revealed significantly higher STAT3 activation scores in both triple-negative and HER2-enriched tumors when compared to luminal tumors (Figure 1F); TrkA activation is not significantly different between subtypes (Figure 1G). However, combined STAT3–TrkA activation signature is highest in TNBC (Figure 1H). Since luminal breast cancers can be divided into A (LumA) and B (LumB) subtypes while triple-negative breast cancers can be divided into claudin-low (CL; mesenchymal) and basal subtypes, we further analyzed the GEO dataset and found similar results (Supplementary Figure S1C–E). To determine whether our findings are relevant in other cancers, we performed GSEA using 592 colorectal cancer patient data as they frequently express oncogenic *NTRK1* fusions [36] and found significant enrichment of STAT3 activation gene signature in patients with high *NTRK1* mRNA levels (Supplementary Figure S2), suggesting that the signaling crosstalk may also be found in other malignant tissues. Taken together, the results in Figure 1 and Figures S1 and S2 demonstrated, for the first time, that JAK2–STAT3 and TrkA pathways are frequently co-activated in triple-negative and HER2-enriched breast cancers.

### 2.2. STAT3 and TrkA Proteins Directly Interact in Triple-Negative and HER2-Enriched Breast Cancers 

**Figure 2:**
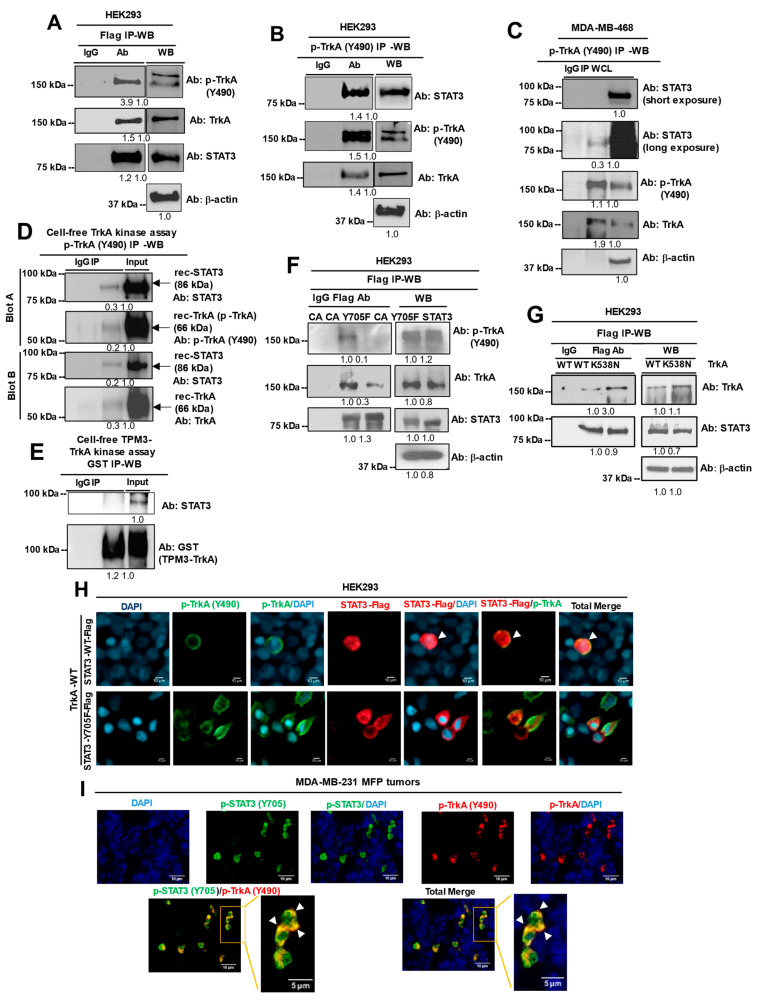
STAT3 and TrkA directly interact in cells and breast tumor tissue. (**A**) IP–Western blot (WB) in HEK293 cells transfected with a STAT3-Flag plasmid. Flag antibody (Ab) was used in IP. (**B**) Reciprocal IP-WB using p-TrkA Ab for IP. (**C**) IP–WB in MDA-MB-468 cells using p-TrkA (Y490) Ab for IP. (**D**) IP–WB of recombinant human TrkA and STAT3 following a cell-free TrkA kinase assay. (**E**) IP–WB of recombinant human TPM3–TrkA and STAT3 following cell-free kinase assay. (**F**) IP–WB of lysates from HEK293 cells transfected with STAT3-CA-Flag or STAT3-Y705F-Flag plasmids. Flag Ab was used in IP. (**G**) IP–WB of lysates from HEK293 cells co-transfected with STAT3-WT-Flag and either TrkA-WT or TrkA-K538N (kinase-dead). Densitometry values are displayed below each Western blot. Original Western blots are found in Supplementary Figures S4–S8. (**H**) Representative IF–confocal microscopy of HEK293 cells co-transfected with a TrkA-WT plasmid plus either a STAT3-WT-Flag or STAT3-Y705F-Flag plasmid, and stimulated with β-NGF for 20 min. DAPI, nuclei (blue). p-TrkA, green. p-STAT3, red. Merged signals, arrows. Scale bar, 10 μm. (**I**) IF staining of a MDA-MB-231 mammary fad xenograft (MFP) tumor for p-STAT3 (green) and p-TrkA (red). DAPI, nuclei (blue). Merged signals, arrows. Scale bars are 10 μm, 5 μm in magnified images.

The observation of co-overexpression of p-STAT3 and p-TrkA prompted us to examine if the co-expression leads to their physical interaction. Of note, these two proteins have never been reported to physically interact. For this, we conducted immunoprecipitation (IP) followed by Western blot using HEK293 cells transfected with flag-tagged STAT3 (STAT3-WT-Flag) and found an interaction between STAT3 and p-TrkA/total TrkA (Figure 2A). The interaction was confirmed in reciprocal IP using a p-TrkA antibody (Figure 2B). To determine whether the STAT3–TrkA interaction occurs in breast cancer cells, we immunoprecipitated endogenous p-TrkA (Y490) from MDA-MB-468 TNBC cells, and Western blot results confirm that endogenous STAT3 co-immunoprecipitates with p-TrkA (Figure 2C). A cell-free TrkA kinase assay followed by immunoprecipitation of p-TrkA reveals that recombinant STAT3 co-immunoprecipitates with p-TrkA, suggesting that these two proteins directly interact (Figure 2D). TrkA undergoes oncogenic fusions in certain cancer types but rarely in breast cancer [18]. According to our datamining of 8767 breast cancer samples (using cBioPortal), only one sample was found to express the TrkA fusion (1/8767). Nevertheless, we next determined whether TrkA fusions also interact with STAT3. To address this, we performed a cell-free kinase assay using GST-tagged Tropomyosin 3 (TPM3)–TrkA fusion protein and recombinant human STAT3, which is followed by immunoprecipitation using an anti-GST antibody. Western blot analysis reveals that STAT3 does not immunoprecipitate with TPM3–TrkA fusion protein (Figure 2E). Next, we asked whether the interaction between STAT3 and p-TrkA is dependent on the phosphorylation of STAT3 on its Y705 residue. To address this question, we compared constitutively active STAT3 (CA) with non-phosphorylation STAT3 mutant (Y705F) for their interaction with p-TrkA using IP-Western blot. The result showed that the STAT3-Y705F mutant lost the ability to interact with p-TrkA, indicating that the Y705 residue is required for the STAT3–TrkA interaction (Figure 2F). Interestingly, these data suggested that TrkA without Y490 phosphorylation can weakly interact with the STAT3-Y705F mutant (Figure 2F). To confirm whether TrkA kinase activity is critical for its interaction with STAT3, we immunoprecipitated flag-tagged STAT3 in HEK293 cells co-transfected with either TrkA-WT or TrkA-K538N (kinase-dead) [37]. Western blot analysis reveals that both TrkA-WT and TrkA-K538N (kinase-dead) mutant co-immunoprecipitate with STAT3, indicating that TrkA kinase activity is not critical for its interaction with STAT3 (Figure 2G).

To confirm the novel STAT3–TrkA interaction and the requirement of STAT3-Y705 for the interaction, we conducted immunofluorescent (IF) staining–confocal microscopy on HEK293 cells co-transfected with TrkA and either STAT3-WT-Flag or STAT3-Y705F-Flag plasmids. To induce phosphorylation of both STAT3 and TrkA, transfected cells were stimulated with human epidermal growth factor (hEGF) and human β-nerve growth factor (β-NGF), respectively. Results of these experiments (Figure 2H, top row) showed that p-TrkA co-localized with STAT3-WT-Flag on or close to the inner cell membrane, as indicated by the yellow merged signals (arrows) from p-TrkA (green) and STAT3-Flag (red). In contrast, p-TrkA failed to co-localize with STAT3-Y705F-Flag, which supports the IP–Western blot finding (Figure 2H, bottom row). Furthermore, we asked whether the STAT3–TrkA interaction can be detected in a triple-negative human breast cancer xenograft. For this, we performed IF staining and confocal microscopy to detect p-STAT3 and p-TrkA in in MDA-MB-231 mammary fat pad (MFP) tumors, and the result showed significant co-localization of both proteins as indicated by the yellow merged signal (arrows) (Figure 2I). Taken together, results in Figure 2 uncovered a novel physical interaction between STAT3 and TrkA proteins, and that their interaction is dependent on STAT3-Y705 phosphorylation.

### 2.3. TrkA Phosphorylates STAT3 at Y705

**Figure 3:**
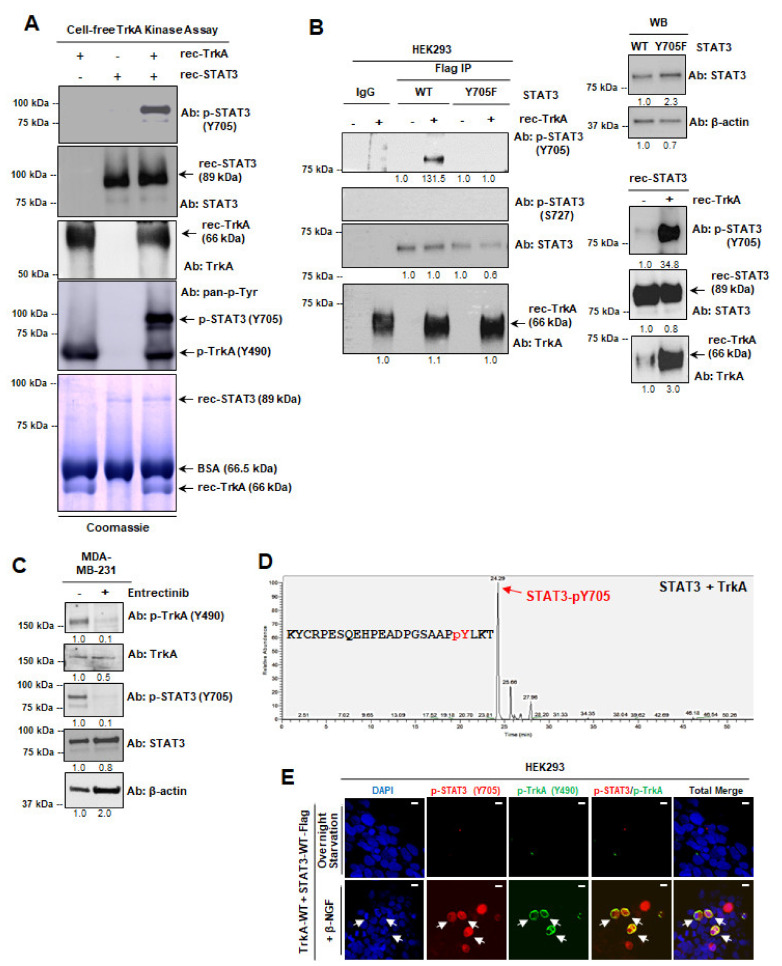
TrkA phosphorylates STAT3 at Y705. (**A**) Cell-free TrkA kinase assay followed by WB to detect p-STAT3 (Y705), total STAT3, total TrkA, and total phosphotyrosines (p-Tyr). Recombinant human STAT3 and TrkA proteins were used. Coomassie-stained gel serves as a loading control for recombinant proteins used in each condition (arrows). (**B**) Immunoprecipitated STAT3-WT-Flag or STAT3-Y705F-Flag proteins were subjected to the TrkA kinase assay, followed by WB to detect p-STAT3 (Y705 or S727). Recombinant proteins are indicated with arrows. (**C**) Western blot of MDA-MB-231 TNBC cells upon treatment with Entrectinib. Densitometry values are displayed below each Western blot. Original Western blots are found in Supplementary Figures S9–S11. (**D**) LC/MS analysis of recombinant human STAT3 after TrkA kinase to detect p-STAT3 (Y705) phosphopeptides. (**E**) Representative IF–confocal microscopy showed that β-NGF induced TrkA activation, as indicated by p-TrkA signals (green), and STAT3 activation, as indicated by nuclear p-STAT3 (red). Scale bars, 10 μm.

Since our data showed that phosphorylated TrkA can interact with STAT3 and the interaction is abolished when STAT3-Y705 is mutated, we hypothesized that TrkA phosphorylates STAT3 at its Y705 residue. While TrkA activation has been observed to promote the phosphorylation of STAT3 on Y705 and S727 residues [27,28] and the pharmacological inhibition of TrkA leads to reduction of STAT3 Y705 phosphorylation [38,39], TrkA phosphorylation of STAT3-Y705 has never been reported. To test this hypothesis, we first conducted a cell-free TrkA kinase assay using recombinant (rec) human TrkA and STAT3 proteins. Kinase reactions were analyzed using Western blot; results showed that TrkA phosphorylates STAT3 at Y705 (Figure 3A). To validate the results of cell-free kinase assay, we immunoprecipitated STAT3 and STAT3-Y705F from transfected HEK293 cells and subjected the immunoprecipitates to the cell-free TrkA kinase assay and Western blot. We found that immunoprecipitated STAT3, but not STAT3-Y705F, can be phosphorylated by TrkA (Figure 3B). We did not detect any signal from an antibody against p-STAT3 (S727), confirming that TrkA kinase specifically targets STAT3-Y705 for phosphorylation. To confirm that the induction of STAT3-Y705 phosphorylation is indeed due to TrkA kinase activity, we treated MDA-MB-231 TNBC cells with TrkA inhibitor Entrectinib. Our Western blot analysis shows significant reduction of p-TrkA (Y490) and concomitant reduction of p-STAT3 (Y705), supporting our hypothesis that TrkA kinase activity is required for STAT3 phosphorylation at Y705 (Figure 3C). To further validate TrkA phosphorylation of STAT3 at Y705, we used liquid chromatography/mass spectrometry (LC/MS) analysis to identify phosphopeptides within the recombinant human STAT3 protein that have been phosphorylated by recombinant TrkA kinase; the results showed the highest enrichment of p-STAT3 (Y705) peptide (Figure 3D; arrow). To determine if TrkA ligand, β-NGF, can induce STAT3 phosphorylation and nuclear transport, we conducted IF followed by confocal microscopy and we showed (Figure 3E) that β-NGF induced TrkA activation, as indicated by p-TrkA signals (green), and STAT3 activation as indicated by nuclear p-STAT3 (red). Together, these results show, for the first time, that TrkA phosphorylates STAT3 and STAT3-Y705 is targeted by TrkA. 

### 2.4. TrkA Phosphorylation Promotes STAT3 Nuclear Import and Transcriptional Activity and Breast Cancer Stem Cells

**Figure 4:**
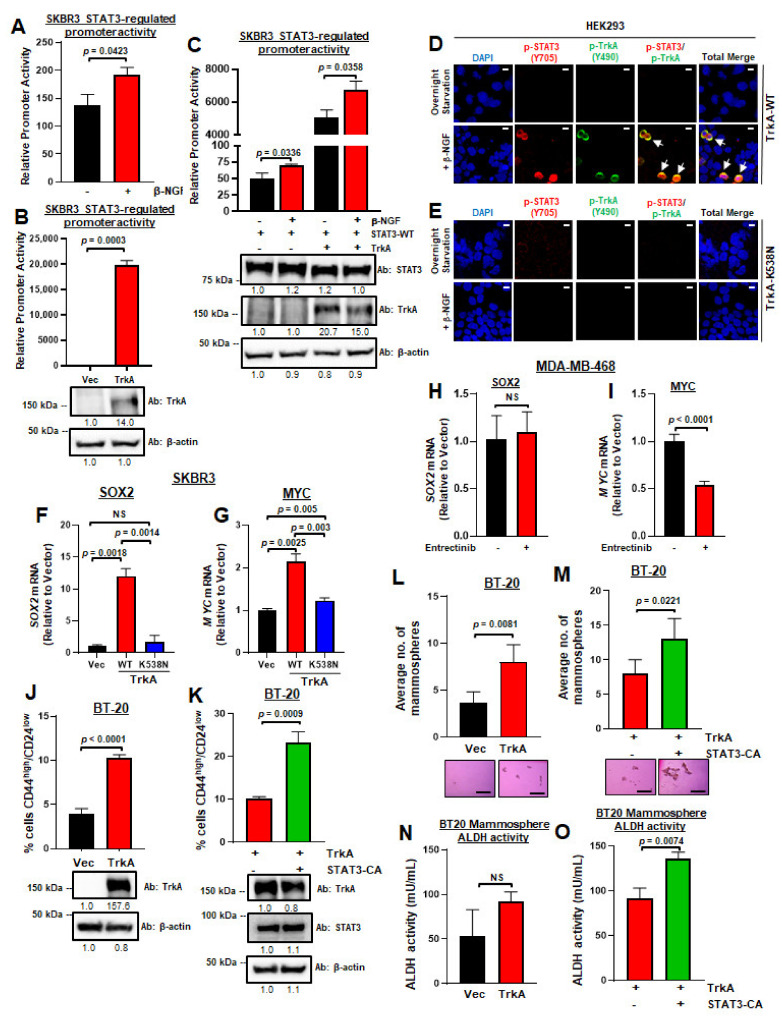
TrkA phosphorylation promotes STAT3 nuclear import and transcriptional activity, and breast cancer stem cells. (**A**) β-NGF stimulation of untransfected SKBR3 breast cancer cells enhances STAT3 transcriptional activity, as shown by luciferase promoter activity. (**B**) TrkA activates STAT3 transcriptional activity as shown by the luciferase promoter activity. SKBR3 cells transfected with TrkA-WT or control vector plus pGAS-Luc (a luciferase reporter under the control of STAT3 response elements) were examined for luciferase activity. (**C**) β-NGF induces pGAS-Luc activity in the presence of TrkA STAT3 or TrkA-STAT3 overexpression. SKBR3 cells were transfected with STAT3-WT plus pGAS-Luc plasmids and with or without the TrkA plasmid, serum-starved, stimulated cells with or without β-NGF for 20 min. and analyzed for luciferase activity. SKBR3 cells transfected with control vector, TrkA-WT, STAT3, or TrkA–STAT3 plasmid plus pGAS-Luc were serum-starved the cells, stimulated with β-NGF for 20 min, and examined for luciferase activity. (**D**,**E**) Representative IF-confocal microscopy showed β-NGF induced activation of STAT3 in the presence of TrkA (**D**), but not kinase-dead TrkA-K538N mutant (**E**). HEK293 cells were used. Merged signals, arrows. Scale bars (upper right), 10 μm. (**F**,**G**) RT-PCR showed that TrkA-WT but not TrkA-K538N induces expression of STAT3 target genes, *SOX2* (**F**) and *MYC* (**G**). SKBR3 were used. (**H**,**I**) RT-PCR of *SOX2* (**H**) and *MYC* (**I**) in MDA-MB-468 upon treatment with a TrkA inhibitor Entrectinib. (**J**,**K**) Flow cytometry of transfected BT20 cells showed that TrkA-WT increases the CD44^high^/CD24^low^ population of breast cancer cells (**J**) and that this effect is enhanced upon the addition of constitutively active STAT3 (**K**). (**L**,**M**) TrkA-WT (**L**) enhances the mammosphere-forming ability of BT20 cells, which is further increased upon the addition of constitutively active STAT3 (**M**). Representative mammosphere images are presented below each graph. Scale bars, 500 μm. (**N**,**O**) Representative ALDH activity of BT20 mammospheres upon transfection with TrkA alone (**N**) or TrkA and constitutively active STAT3 (**O**). All experiments were done for at least three times. Student’s t-test were used to determine *p*-values; NS: not significant. Data represented as mean ± SD. Western blots and densitometry values for each transfection condition are displayed below each plot. Original Western blots are found in Supplementary Figures S12 and S14.

STAT3 phosphorylation at Y705 leads to STAT3 nuclear translocation and activation of transcriptional activity. Previous studies have shown that STAT3-mediated transcriptional activity can be enhanced by the addition of TrkA ligand β-NGF [28] or overexpression of Trk fusion genes [27]; however, whether it is occurred directly through TrkA-mediated STAT3 activation has remained unclear. Here, we tested whether TrkA-mediated phosphorylation of STAT3 at Y705 activates the ability of STAT3 to under nuclear transport and activate a STAT3-targeted promoter. For this, we transfected HER2-enriched SKBR3 cells (with low endogenous TrkA and STAT3) with TrkA-WT or control vector plus pGAS-Luc (a luciferase reporter under the control of STAT3 response elements) and determined luciferase activity. As shown in Figure 4A, β-NGF stimulation of untransfected SKBR3 breast cancer cells following starvation induced significant upregulation of pGAS-Luc activity, suggesting that the activation of endogenous TrkA can induce STAT3 nuclear translocation and transcriptional activity. Accordingly, TrkA overexpression strongly activated pGAS-Luc activity indicating TrkA activates STAT3 transcriptional activity, which is in agreement with previous reports (Figure 4B) [27]. To determine whether TrkA ligand β-NGF induces pGAS-Luc and if TrkA and STAT3-WT co-induces pGAS-Luc activity, we co-transfected SKBR3 cells with STAT3-WT and pGAS-Luc plasmids (with or without the TrkA plasmid), serum-starved the cells, and stimulated cells with or without β-NGF, and determined luciferase activity. As shown in Figure 4C, β-NGF induced pGAS-Luc activity in the presence of STAT3 or TrkA-STAT3. Importantly, cells with TrkA-STAT3 co-transfection and β-NGF stimulation had the highest pGAS-Luc activity. 

To determine if β-NGF-induced activation of STAT3 is attributed to TrkA kinase activity, we examined TrkA-WT versus TrkA-K538N (dominant-negative kinase-dead mutant) [37] in their ability to induce STAT3 Y705 phosphorylation and nuclear transport. HEK293 cells were co-transfected with STAT3-WT-Flag and either TrkA–WT (Figure 4D) or TrkA-K538N (Figure 4E), starved for serum for 16 h, stimulated with β-NGF, and examined for STAT3 Y705 phosphorylation and nuclear import using IF staining-confocal microscopy. As expected, neither p-TrkA nor p-STAT3 was detected in serum-starved cells. Stimulation with β-NGF activated TrkA, as indicated by the detection of p-TrkA (green signals) at the cell membrane (Figure 4D). Importantly, β-NGF induced p-STAT3, which was detected in the cytosol as well as in the nucleus, indicating that TrkA activation induced STAT3 Y705 phosphorylation and nuclear translocation (yellow merge signals, arrows; Figure 4D). In contrast, no phosphorylation events, in either TrkA or STAT3, were detected in cells overexpressing the kinase-dead TrkA-K538N mutant (Figure 4E). 

Activated STAT3, a transcription factor, can induce the transcription of many target genes implicated in malignant progression, some of which are involved in cell proliferation, survival, invasion, and metastasis [40]. To further determine if STAT3 transcriptional activity can be activated through TrkA, we performed RT-PCR analysis of representative STAT3 target genes using SKBR3 cells overexpressing TrkA-WT or TrkA-K538N. Results showed that TrkA WT-overexpressing cells had significantly higher mRNA levels of *SOX2* (Figure 4F) and *MYC* (Figure 4G), which are two known STAT3 target genes frequently associated with breast cancer cell stemness and aggressiveness [41,42]. Interestingly, cells overexpressing TrkA-WT also showed a modest increase in *STAT3* mRNA expression, which is abrogated in TrkA-K538N expressing cells (Supplementary Figure S13A,B), suggesting that TrkA can regulate STAT3 expression as well as STAT3 transcriptional activity. Conversely, *SOX2* or *MYC* mRNAs were significantly downregulated in cells expressing TrkA-K538N, nearly to the levels found in the vector controls, suggesting that TrkA kinase activity is essential for inducing STAT3 target gene transcription in breast cancer cells. To confirm that the increase in *SOX2* and *MYC* gene expression is indeed due to TrkA kinase activity, we treated MDA-MB-468 TNBC cells, which have high levels of p-TrkA (Y490) (Figure 1C), with a TrkA inhibitor Entrectinib and performed RT-PCR for *SOX2* and *MYC*. We found that while *SOX2* expression was not altered upon TrkA inhibition (Figure 4H), *MYC* expression is significantly downregulated in the absence of TrkA activity (Figure 4I). It is important to note that treatment with Entrectinib does not impact *NTRK1* mRNA (Supplementary Figure S13C); however, there is a significant reduction in *STAT3* expression upon treatment with Entrectinib (Supplementary Figure S13D), which is in agreement with our results that TrkA kinase activity may also regulate STAT3 expression in addition to STAT3 activity (Supplementary Figure S13B). To confirm whether TrkA-mediated STAT3 activation can indeed promote breast cancer cell stemness, we transiently overexpressed TrkA in BT20 cells, which have relatively lower levels of activated TrkA and STAT3, with and without constitutively active STAT3 (STAT3-CA), and we performed flow cytometry to determine changes in the CD44^high^/CD24^low^ population of cells. Relative to vector controls, TrkA-overexpressing BT20 cells had significantly higher percentage of CD44^high^/CD24^low^ cells (4% vs. 10.3%, respectively) (Figure 4J). Compared to TrkA alone, the addition of STAT3-CA further enhanced the effect of TrkA in promoting the CD44^high^/CD24^low^ population (10.3% vs. 23.3%), which supports the signaling crosstalk between JAK2/STAT3 and TrkA pathways (Figure 4K). Then, we performed mammosphere assays on BT20 cells transiently overexpressing TrkA, with and without STAT3-CA. Compared to vector controls, TrkA-overexpressing BT20 cells formed significantly higher number of mammospheres (Figure 4L). The addition of STAT3-CA enhanced the effect of TrkA on the mammosphere-forming ability of BT20 cells and yielded the highest number of mammospheres, which is reflected in the representative images (Figure 4M). To further validate the induction of cancer cell stemness in breast cancer cells overexpressing TrkA, we performed an aldehyde dehydrogenase (ALDH) assay in BT20 TNBC cells after transient transfection with TrkA, with and without STAT3-CA. The induction of ALDH activity is a marker for cancer cell stemness and may be predictive of poor clinical outcome in breast cancer [43]. Our ALDH assay results indicated that TrkA can increase ALDH activity in BT20 breast cancer cells when compared to vector controls (Figure 4N), although this did not reach statistical significance. However, in BT20 cells overexpressing TrkA, STAT3-CA significantly increased ALDH activity (Figure 4O). Taken together, these data suggest that TrkA-mediated phosphorylation and activation of STAT3 can induce STAT3 nuclear transport and the transcription of STAT3 target genes that promote breast cancer stem cells. 

### 2.5. Co-Activation of JAK2–STAT3 and TrkA Pathways Is Correlated with Poor Overall and Bone Metastasis-Free Survival of Triple-Negative and HER2-Enriched Breast Cancers

**Figure 5:**
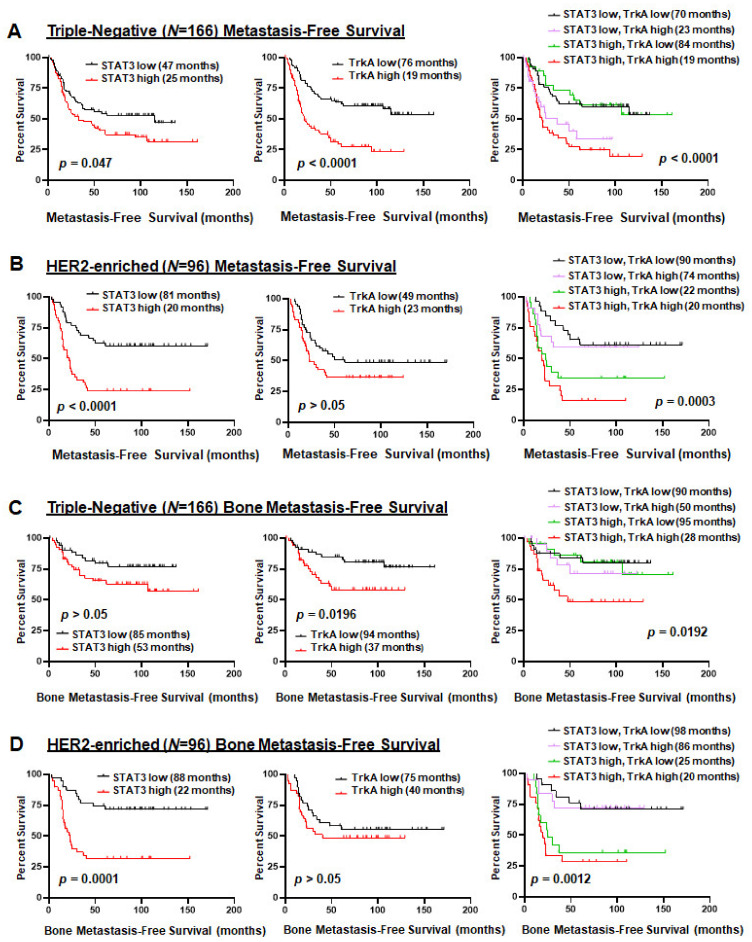
Co-activation of JAK2–STAT3 and TrkA pathways is correlated with poor overall and bone metastasis-free survival of triple-negative and HER2-enriched breast cancers. Using Kaplan–Meier analysis log-rank test, and the STAT3 or TrkA activation signatures, we analyzed patients whose expression profiles and survival data were obtained from GEO databases (GSE 2034, 2603, 5327, 12,276). (**A**) Overall metastasis-free survival in 166 triple-negative breast cancer. (**B**) Overall metastasis-free survival in 96 HER2 breast cancer. (**C**) Bone metastasis-free survival in 166 triple-negative breast cancer patients. (**D**) Bone metastasis-free survival in 96 HER2 breast cancer patients. Median survival times are indicated.

We have shown that activated STAT3 and activated TrkA can be found in triple-negative and HER2-enriched breast cancers and that the combined JAK2–STAT3/TrkA activation signatures are significantly enriched in these two subtypes (Figure 1). Since triple-negative and HER2-positive breast cancers are more aggressive than the luminal subtype and have higher rates of metastasis, we examined GEO datasets to determine if JAK2–STAT3 and TrkA pathway activation, individually and jointly, is associated with overall metastasis-free survival in patients with these two tumor subtypes. In 166 TNBC patients, we found tumors with high STAT3 activation, high TrkA activation, and co-activation to correlate with a shortened time to develop overall metastasis (Figure 5A). In 96 HER2-enriched breast cancer patients, we observed that high STAT3 activation and high JAK2-STAT3/TrkA co-activation, but not high TrkA activation, correlate with a shortened time to develop overall metastasis (Figure 5B). Furthermore, in TNBC, we found that high TrkA activation and high co-activation, but not high STAT3 activation, were associated with a shortened time to develop bone metastasis (Figure 5C). In HER2-enriched breast cancer, high STAT3 activation and high co-activation, but not high TrkA activation, correlated with an increase potential to develop bone metastasis (Figure 5D). Together, these data indicate the novel prognostic value for the co-activation of JAK2–STAT3 and TrkA for metastatic triple-negative and HER2-enriched breast cancers. 

### 2.6. Co-Activation of JAK2-STAT3 and TrkA Pathways Is Associated with a Shortened Time to Develop Brain and Lung Metastasis of Triple-Negative and HER2-Enriched Breast Cancers

**Figure 6:**
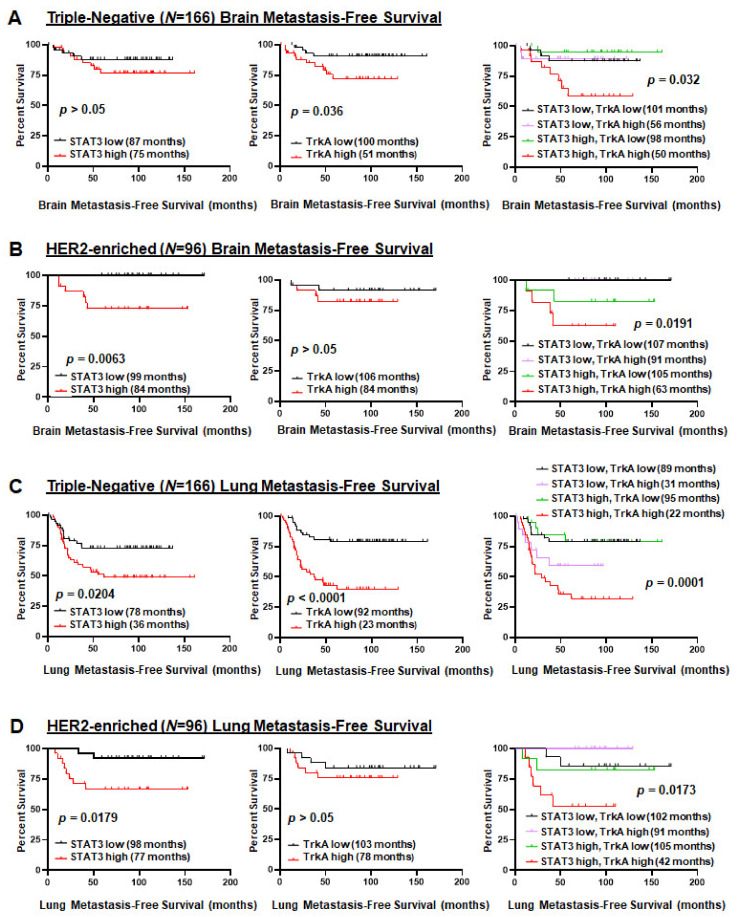
Co-activation of JAK2-STAT3 and TrkA pathways is associated with poor brain- and lung metastasis-free survival of triple-negative and HER2-enriched breast cancers. Using Kaplan–Meier analysis log-rank test, and the STAT3 or TrkA activation signatures, we analyzed patients whose expression profiles and survival data were obtained from GEO databases (GSE 2034, 2603, 5327, 12,276). (**A**) Brain metastasis-free survival in 166 triple-negative breast cancer patients. (**B**) Brain metastasis-free survival in HER2 breast cancer. (**C**) Lung metastasis-free survival in triple-negative breast cancer. (**D**) Lung metastasis-free survival in 96 HER2-enriched breast cancer patients. Median survival times are indicated.

Further survival analyses of triple-negative breast cancer datasets showed that high TrkA activation and high co-activation, but not STAT3 activation, were associated with shortened time to develop brain metastasis (Figure 6A). For HER2-enriched breast cancer (*N* = 96), we found that high STAT3 activation and high co-activation, but not TrkA activation, were associated with a shortened time to develop brain metastasis (Figure 6B). For lung metastasis-free survival of triple-negative breast cancer (*N* = 166), we observed that high STAT3 activation, high TrkA activation, and high co-activation, were associated with a high likelihood to develop lung metastasis (Figure 6C). For lung metastasis-free survival of HER2-enriched breast cancer, high STAT3 activation and high co-activation, but not TrkA activation, were associated a higher potential to develop lung metastasis (Figure 6D). Collectively, the results in Figure 6 indicate the novel prognostic value for the co-activation of JAK2–STAT3 and TrkA for brain- and lung-metastatic triple-negative and HER2-enriched breast cancers.

## 3. Discussion

In this study, we have made the following novel and impactful findings. (1) JAK2–STAT3 and TrkA pathways are significantly co-activated in triple-negative and HER2-positive human breast cancer specimens. (2) STAT3 and TrkA proteins interact directly as observed in cells and MFP xenograft tissues. (3) The STAT3–TrkA interaction is dependent on STAT3 Y705. (4) TrkA phosphorylates STAT3 at Y705 to induce its activation, nuclear import, and transcriptional activation of known STAT3 target genes, *SOX2* and *MYC*. (5) Co-activation of TrkA and STAT3 promotes breast cancer stem cells. (6) JAK2–STAT3 and TrkA pathway co-activation is significantly higher in triple-negative and HER2-enriched breast cancers when compared to luminal subtypes of cancers. (7) High JAK2–STAT3 and TrkA co-activation in TNBC and HER2-enriched breast cancers is associated with shorter metastasis-free survival and significantly higher risks for lung, bone, and brain metastasis. Through these significant observations, our study uncovers TrkA-mediated STAT3 phosphorylation as a novel mechanism underlying the crosstalk between the JAK2–STAT3 and TrkA pathways in triple-negative and HER2-enriched breast cancers, and it identifies the novel prognostic value for the pathway co-activation. 

While previous reports showed TrkA activation can lead to STAT3 phosphorylation [27,28] and that the inhibition of TrkA can abrogate STAT3 phosphorylation [38,39], the current study is the first to show that TrkA directly and physically interacts with STAT3 and that TrkA is a novel upstream kinase of STAT3. TrkA-mediated phosphorylation and activation of STAT3 represents a novel mechanism by which STAT3 is activated in human cancers. While JAK2 has been regarded as the key tyrosine kinase responsible for phosphorylating STAT3 at Y705, it has been reported that loss of JAK2 does not significantly affect STAT3 activation [44], suggesting that STAT3 phosphorylation and activation can be sustained independently of JAK2 activity. It has also been shown that JAK1 plays an important role in STAT3 activation in breast cancer [45]. Since all three kinases target Y705 of STAT3, it would be important to determine and compare the ability of these three STAT3-activating kinases in binding to STAT3 and phosphorylating STAT3. 

Since STAT3 serves as a converging point of several oncogenic signaling pathways, including JAK1/2, EGFR, HER2, Src [40], and TrkA (current study) and STAT3 transcriptionally activates expression of many important cancer-related genes [40], it is critically important to completely inhibit STAT3 activity to effectively inhibit cancer growth and metastasis. In this regard, combined inhibition of multiple STAT3-activating kinases should be exploited. To facilitate the testing of these combination treatments, it is an important task to determine the extent to which these STAT3-activating kinases are co-activated in breast cancers. It is also imperative to examine if there is any cooperation among these kinases. 

Given the observed co-activation of JAK2 and TrkA in triple-negative and HER2-enriched breast cancers, we speculate that co-targeting of JAK2 and TrkA would be a promising new therapeutic modality against breast cancer. Currently, there are two FDA-approved orally active TrkA inhibitors, entrectinib and larotrectinib. Entrectinib (Rozlytrek^®^) was approved in August 2019 for treatment of solid tumors that bore oncogenic *NTRK1* fusions. Larotrectinib (Vitravki^®^) was approved in 2018 as a tissue-agnostic inhibitor for any solid tumors bearing *NTRK1* fusions [25,26]. There are three FDA-approved JAK2 inhibitors, ruxolitinib, baricitinib, and fedratinib [15,16,17]. Ruxolitinib (also known as Jakafi^®^) was approved in 2011 for treatment of myelofibrosis and is currently in Phase 1 and 2 clinical trials for treating breast cancer patients. Baricitinib (Olumiant^®^) is approved for the treatment of rheumatoid arthritis and is not being evaluated for breast cancer. Fedratinib (Inrebic^®^) is approved for treating primary and secondary myelofibrosis; however, it has not entered clinical trials for breast cancer patients. Additional JAK1/2 inhibitors, pacritinib, gandotinib, lestaurtinib, and momelotinib, are in clinical trials for myelofibrosis, myeloid neoplasms, or relapsed lymphomas, but they have not yet been investigated clinically in breast cancer patients [46,47,48,49].

We also observed an increase in the transcription of STAT3 target genes, *SOX2* and *MYC*, upon the overexpression of TrkA (Figure 4F,G). *SOX2* is critical for maintaining the pluripotency of embryonic stem cells and has been credited with the induction and maintenance of breast cancer cell stemness [50,51]. The induction of breast cancer cell stemness is frequently implicated in breast cancer recurrence as well as therapeutic resistance [52]. Indeed, *SOX2* expression is adequate to induce mammosphere formation and promotes tumor growth in a xenograft model of breast cancer [50]. In addition to promoting cell proliferation, *MYC* overexpression has also been implicated in breast cancer cell stemness. Yin et al. and other groups show that the overexpression of *MYC* in triple-negative breast cancer cells can induce the expression of ALDH1 protein, which is considered a stem cell marker in a number of solid tumors, resulting in higher rates of stem cell self-renewal [53,54]. In agreement with these findings, we show that TrkA-mediated induction breast cancer cell stemness and mammosphere-forming ability can be enhanced by STAT3, further supporting a cooperative signaling crosstalk between the TrkA and JAK2/STAT3 pathways (Figure 4L,M). Thus, TrkA-mediated activation of STAT3 can induce the expression of stemness genes to support breast cancer progression. Whether the downregulation of both JAK2–STAT3 and TrkA signaling can suppress breast cancer cell stemness remains to be examined. 

In summary, our study reports that TrkA is a novel upstream activating kinase for STAT3, the TrkA and JAK2 pathways are concurrently activated in triple-negative and HER2-positive breast cancers, and the co-activation serves as a contributing factor to breast cancer stem cells and a new prognosis indicator for breast cancer metastasis to various organs. Together, our findings shed new light into the pathobiology of breast cancer with concurrent expression of TrkA and STAT3. Our observations also provide first evidence to support future testing of a novel combination therapy for metastatic triple-negative and HER2-enriched breast cancers through simultaneous inhibition of JAK2 and TrkA kinase activities.

## 4. Materials and Methods 

### 4.1. Cell Culture, Cell Lines, and Reagents

All human cell lines were purchased from the American Type Culture Collection (Manassas, VA, USA). The breast cancer cell lines used in this study included triple-negative MDA-MB-231, MDB-MB-468, and BT20 cells, as well as LumB HER2-amplified SKBR3 cells. Cells were maintained in Corning DMEM (high glucose with sodium pyruvate; Corning Incorporated, Corning, NY, USA) or MEM (ThermoFisher Scientific, Waltham, MA, USA) supplemented with 10% heat-inactivated fetal bovine serum (Gibco, Gaithersburg, MD, USA) and 1% penicillin/streptomycin. 

### 4.2. Immunohistochemistry (IHC) and Tumor Scoring

Breast cancer tissue microarray (BR10010e) was purchased from US Biomax (Rockville, MD, USA) and stained for p-TrkA (Y490) and p-STAT3 (Y705) as previously described [55,56]. IHC scoring was performed as previously described [55]. Briefly, histologic scores (H-scores) were computed from both percent positivity (A%, A = 1–100) and intensity (B = 0–3) using the following equation: H-score = A × B. For p-TrkA (Y490) and p-STAT3 (Y705) H-score, tumors were classified as low (H-score = 0–150) or high (H-score = 150–300). 

### 4.3. Plasmids and Transfection

pCMV5-TrkA was generated by Raymond Birge (Addgene #15002, Watertown, MA, USA) [57]. pLEGFP-Y705F-STAT3 was made by George Stark (Addgene #71445) [58]. pLEGFP-STAT3-WT-Flag was generated by performing site-directed mutagenesis on the Y705F mutant plasmid to restore Y705 using Quik-Change II XL mutagenesis kit (Agilent Technologies, Santa Clara, CA, USA). Constitutively activate STAT3 (STAT3-CA) was generously gifted by Keping Xie at the University of Texas-MD Anderson Cancer Center [59]. pCMV5-TrkA-K538N kinase-dead mutant was generated by performing site-directed mutagenesis on the wild-type plasmid. Mutagenesis primers are described in Supplementary Table S1. All transfections were performed using XtremeGene HP transfection reagent (Roche, Basel, Switzerland) and OPTI-MEM I Reduced Serum Medium (Gibco) as per manufacturers’ protocols.

### 4.4. Metastasis-Free Survival, Gene Set Enrichment Analyses (GSEA), and Statistical Analysis

Publicly available breast tumor RNA-Seq expression profiles were retrieved from TCGA and GEO (GSE 2034, 2603, 5327, 12,276) with subtype and/or metastasis-free survival information from 166 triple-negative breast cancer patients and 96 HER2-enriched breast cancer patients. Median centering was used to generate STAT3 or TrkA activation signature comprised of known genes regulated by STAT3 [35] or TrkA (R-HSA-187037) [34] pathway activity. For Kaplan–Meier survival analyses, STAT3 or TrkA activation scores were calculated, and patients were stratified 50:50 (high vs. low activation) based on the median activation score and plotted using GraphPad Prism 8. The Log-rank test was used to determine significance. GSEA was performed by generating the Gene MatriX file (.gmx) using published gene signatures for STAT3 and/or TrkA activation [29,60,61]. Gene Cluster Text files (.gct) and Categorical Class files (.cls) were generated based on STAT3 and/or TrkA score in the GEO datasets. The number of permutations for GSEA was set to 1000 and the GEO gene list was used as the chip platform. TrkA fusion gene (TFG) mRNA was analyzed from TCGA breast cancer datasets. Results are represented as mean ± SD. Log-rank test and Tukey’s test were performed using GraphPad Prism 8 (GraphPad Software, San Diego, CA, USA).

### 4.5. Immunoprecipitation (IP) and Western Blot 

For crude protein lysates, adherent cells were washed with cold 1× PBS before lysis with RIPA buffer (ThermoFisher Scientific) or SDS-free RIPA buffer. Lysis buffers were prepared with 1× Halt Protease/Phosphatase inhibitor (ThermoFisher Scientific). Lysates were sonicated and clarified by centrifugation before quantification with Bradford assay (Bio-Rad, Hercules, CA, USA). Flag IP experiments were performed as previously described [62]. To IP p-TrkA (Y490), anti-p-TrkA (Y490) antibody was conjugated to Dynabeads Protein G beads (Invitrogen, Carlsbad, CA, USA) as per manufacturer’s protocol before overnight incubation with crude protein lysates at 4 °C. 

Western blot of IP products was performed as previously described [55]. All antibodies are described in Supplementary Table S2.

### 4.6. Immunofluorescence (IF) Staining and Confocal Microscopy

HEK293 cells were seeded at 1.5 × 10^4^ cells/well into Nunc Lab-Tek II 8-well chamber slide (ThermoFisher) and transfected with the indicated plasmids. Transfected cells were starved overnight before stimulation with 100 ng/mL of β-NGF (R&D Systems, Minneapolis, MN, USA) or human EGF (Sigma Aldrich, St. Louis, MO, USA) for 20 min at 37 °C. Cells were fixed with 4% paraformaldehyde for 30 min at ambient temperature. Fixed cells were washed in 1× PBS before blocking (5% goat serum + 0.3% Triton X-100 in 1× PBS) for 1 h, followed by incubation with primary antibody overnight at 4 °C. Cells were incubated with a fluorophore-conjugated secondary antibody (see Supplementary Table S2) for 1 h at room temperature. Nuclei were stained with DAPI while mounting slides with a coverslip (Vector Labs, Burlingame, CA, USA). Slides were imaged using the Olympus FV1200 confocal microscope (Center Valley, PA, USA) or ImageXpress Micro Confocal System (Molecular Devices, San Jose, CA, USA). 

For MFP tissue immunostaining, 5 µm frozen sections were used. All sections were fixed using cold acetone for 10 min. and blocked with 1× PBS/0.3% Tween/5% goat serum. Sections were stained using mouse anti-phospho-STAT3 (Y705) and rabbit anti-phospho-TrkA (Y490) simultaneously, in 1× PBS/0.3% Tween/5% goat serum overnight at 4 °C. Phospho-STAT3 (Y705) was detected with goat anti-mouse Alexa Fluor 488 (Invitrogen) while phospho-TrkA (Y490) was detected using goat anti-rabbit Alexa Fluor 568 (Invitrogen). Nuclei were labeled with DAPI, and slides were mounted with a cover slip (Vector Labs). Slides were imaged using the Olympus FV1200 confocal microscope, and Z-stack images were acquired and processed with FV10-ASW 4.2 viewer software.

### 4.7. TrkA Cell-Free Kinase Assay, Mass Spectrometry, and IP-Kinase Assay

Recombinant human STAT3 (Creative Biomart, Shirley, NY, USA) was incubated with enzymatically active recombinant human TrkA (Abcam, Cambridge, UK) or TPM3-TrkA (MyBioSource, San Diego, CA, USA) for 30 min at 30 °C in kinase reaction buffer (52 mM Tris, 43 mM MgCl_2_, 1 mg/mL BSA, pH 7.5) in the presence of 50 mM ATP (New England BioLabs, Ipswich, MA, USA). Kinase reactions were terminated by the addition of Laemmli sodium dodecyl sulfate (SDS) buffer and resolved on 8% SDS polyacrylamide gel. For LC/MS analyses, STAT3 bands were excised using sterile blades and submitted for liquid chromatography/mass spectrometry at the Massachusetts Institute of Technology Proteomics Core. For Coomassie staining, gels were fixed (50:40:10/methanol:water:acetic acid) for 1 h, followed by overnight staining with Coomassie R-250 (G-Biosciences, St. Louis, MO, USA) and de-staining until bands of interest were visible. Coomassie-stained gels were imaged using the Bio-Rad ChemiDoc. For IP-kinase assays, HEK293 cells were transfected with flag-tagged wild-type STAT3 or STAT3-Y705F and underwent Flag IP. Flag IP products were subjected to TrkA kinase assay as described and reaction products were analyzed by Western blot. For kinase assay-IP, TrkA cell-free kinase assay was repeated and recombinant active TrkA was immunoprecipitated using anti-TrkA (Y490) antibody, resolved using SDS-PAGE and examined for co-immunoprecipitation of recombinant STAT3 using Western blot. 

### 4.8. Pharmacological Inhibition of TrkA

MDA-MB-231 cells were seeded on a six-well culture dish and allowed to adhere overnight. Cells were treated with 5 μM Entrectinib (Adooq BioScience, Irvine, CA, USA) or 1% DMSO vehicle overnight at 37 °C and immediately harvested for Western blot analysis.

### 4.9. STAT3 Luciferase Assay

Luciferase assays were performed using a dual-luciferase assay kit (Biotium, Fremont, CA, USA) and readouts were collected using the Molecular Devices iD3 plate reader (San Jose, CA, USA). Cells were co-transfected with a STAT3-responsive luciferase reporter (pGAS-luc), and either vector or TrkA (pCMV5-TrkA) plasmid for 28 h before serum starvation for 16 h. Then, cells were stimulated with 100 ng/mL β-NGF for 4 h before the addition of lysis buffer. Lysates underwent mechanical lysis by scraping followed by trituration with a pipette to ensure complete lysis. Firefly luciferase activity was measured as previously described [56,63]. Results are represented as mean ± SD. Student’s t-tests were performed using GraphPad Prism 8.

### 4.10. Quantitative RT-PCR

Total RNA was isolated from cells using QIAGEN RNEasy Mini Kit (Hilden, Germany). RT-PCR was performed as previously described using primers listed in Supplementary Table S3 [62,64].

### 4.11. Flow Cytometry, Mammosphere Assays and ALDH Activity Assay

Flow cytometry for CD44^high^/CD24^low^ cells and mammosphere assays were performed as previously described [65]. For ALDH activity, BT20 cells were transfected for 24 h and seeded in mammosphere conditions for up five days. Mammospheres were then pelleted and subjected to ALDH activity assay using the Abcam colorimetric ALDH activity kit (Cambridge, UK).

## 5. Conclusions

The current study is the first to illustrate that JAK2–STAT3 and TrkA pathways are co-activated in metastatic triple-negative and HER2-enriched breast cancers. Additionally, we show that TrkA interacts with and phosphorylates STAT3 on its Y705 residue, inducing oncogenic gene transcription to support breast cancer cell stemness, and that loss of Y705 residue abrogates STAT3 interaction with TrkA. These findings suggest that JAK2–STAT3 and TrkA signaling pathways converge at STAT3, supporting the crosstalk between these two signaling cascades. Importantly, we show that JAK2–STAT3/TrkA pathway co-activation is a new prognostic indicator of distant metastasis to various organs, prompting a future study to examine combined inhibition of JAK2–STAT3 and TrkA pathways as a novel and viable combination therapy in metastatic triple-negative and HER2-enriched breast cancers.

## Data Availability

No new datasets were generated in this manuscript.

## Supplementary Materials

The following are available online at https://www.mdpi.com/article/10.3390/cancers13102340/s1, Figure S1: Expanded GSEA and datamining analyses of GEO breast cancer datasets using STAT3, TrkA, and combined STAT3–TrkA activation signatures, Figure S2: GSEA of STAT3 activation signature in colorectal cancer (CRC) patients, Figure S3: Original Western blot images for Figure 1C, Figure S4: Original Western blot images for Figure 2A,B, Figure S5: Original Western blot images for Figure 2C, Figure S6: Original Western blot images for Figure 2D,E, Figure S7: Original Western blot images for Figure 2F, Figure S8: Original Western blot images for Figure 2G, Figure S9: Original Western blot images for Figure 3A, Figure S10: Original Western blot images for Figure 3B, Figure S11: Original Western blot images for Figure 3C, Figure S12: Original Western blot images for Figure 4B,C, Figure S13: mRNA levels of *NTRK1* and *STAT3* upon overexpression or inhibition of TrkA, Figure S14: Original Western blot images for Figure 4J,K, Table S1: Site-directed mutagenesis primers, Table S2: Antibodies, Table S3: RT-PCR primer sequences.
