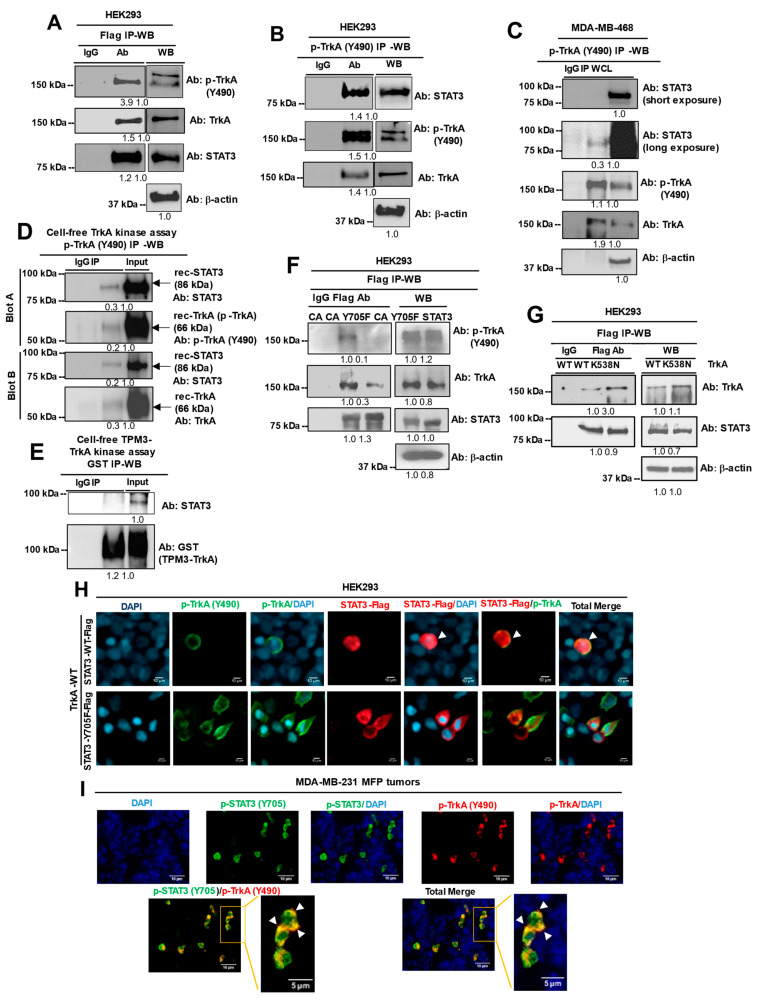# Correction: Regua et al. TrkA Interacts with and Phosphorylates STAT3 to Enhance Gene Transcription and Promote Breast Cancer Stem Cells in Triple-Negative and HER2-Enriched Breast Cancers. *Cancers* 2021, *13*, 2340

**DOI:** 10.3390/cancers16193409

**Published:** 2024-10-08

**Authors:** Angelina T. Regua, Noah R. Aguayo, Sara Abu Jalboush, Daniel L. Doheny, Sara G. Manore, Dongqin Zhu, Grace L. Wong, Austin Arrigo, Calvin J. Wagner, Yang Yu, Alexandra Thomas, Michael D. Chan, Jimmy Ruiz, Guangxu Jin, Roy Strowd, Peiqing Sun, Jiayuh Lin, Hui-Wen Lo

**Affiliations:** 1 Department of Cancer Biology, Wake Forest University School of Medicine, Winston-Salem, NC 27101, USA; aregua@wakehealth.edu (A.T.R.); raguayo@highpoint.edu (N.R.A.); sabujal@wakehealth.edu (S.A.J.); ddoheny@wakehealth.edu (D.L.D.); smanore@wakehealth.edu (S.G.M.); dozhu@wakehealth.edu (D.Z.); glwong@wakehealth.edu (G.L.W.); aarrigo@wakehealth.edu (A.A.); cjwagner@wakehealth.edu (C.J.W.); yayu@wakehealth.edu (Y.Y.); gjin@wakehealth.edu (G.J.); psun@wakehealth.edu (P.S.); 2 Department of Hematology and Oncology, Wake Forest University School of Medicine, Winston-Salem, NC 27101, USA; althomas@wakehealth.edu (A.T.); jruiz@wakehealth.edu (J.R.); 3 Breast Cancer Center of Excellence, Wake Forest University School of Medicine, Winston-Salem, NC 27101, USA; 4 Wake Forest Baptist Comprehensive Cancer Center, Wake Forest University School of Medicine, Winston-Salem, NC 27101, USA; mchan@wakehealth.edu (M.D.C.); rstrowd@wakehealth.edu (R.S.); 5 Department of Radiation Oncology, Wake Forest University School of Medicine, Winston-Salem, NC 27101, USA; 6 Department of Neurology, Wake Forest University School of Medicine, Winston-Salem, NC 27101, USA; 7 Department of Biochemistry and Molecular Biology, University of Maryland School of Medicine, Baltimore, MD 21201, USA; jlin@som.umaryland.edu

## Error in Figure

In the original publication [1], there was a mistake in Figure 2D. IP-WB of recombinant human TrkA and STAT3 following a cell-free TrkA kinase assay as published. 

In the original experiment, the Western blot was performed with one membrane probed for STAT3 (upper membrane) and p-TrkA (lower membrane), followed by the re-probing of the lower membrane for TrkA. We recently became aware that the TrkA bands may have been carryovers from the p-TrkA bands. Therefore, we repeated the same experiment but loaded the immunoprecipitates and input lysates in duplicates to produce two duplicate membranes, allowing us to probe one for p-TrkA and the other for TrkA. The replacement did not change the conclusion of the figure or the study. The corrected Figure 2D appears below. Additionally, we have updated Supplementary Figure S6 to include the original Western blot images for this correction. The authors apologize for any inconvenience caused and state that the scientific conclusions are unaffected. This correction was approved by the Academic Editor. The original publication has also been updated.