# Supplementary material for: TrkA Interacts with and Phosphorylates STAT3 to Enhance Gene Transcription and Promote Breast Cancer Stem Cells in Triple-Negative and HER2-Enriched Breast Cancers
Source: Cancers (Basel). 2021 May 12;13(10):2340. doi: 10.3390/cancers13102340 (PMC8150921; doi:10.3390/cancers13102340)
Supplement: Supplementary file 1 [file cancers-13-02340-s001.zip › cancers-1201378-supplementary.pdf]

Supplementary Materials: TrkA Interacts with and Phosphorylates STAT3 to Enhance Gene Transcription and Promote Breast Cancer Stem Cells in Triple-Negative and HER2-Enriched Breast Cancers

Angelina T. Regua, Noah R. Aguayo, Sara Abu Jalboush, Daniel L. Doheny, Sara G. Manore, Dongqin Zhu, Grace L. Wong, Austin Arrigo, Calvin J. Wagner, Yang Yu, Alexandra Thomas, Michael D. Chan, Jimmy Ruiz, Guangxu Jin, Roy Strowd, Peiqing Sun, Jiayuh Lin, and Hui-Wen Lo

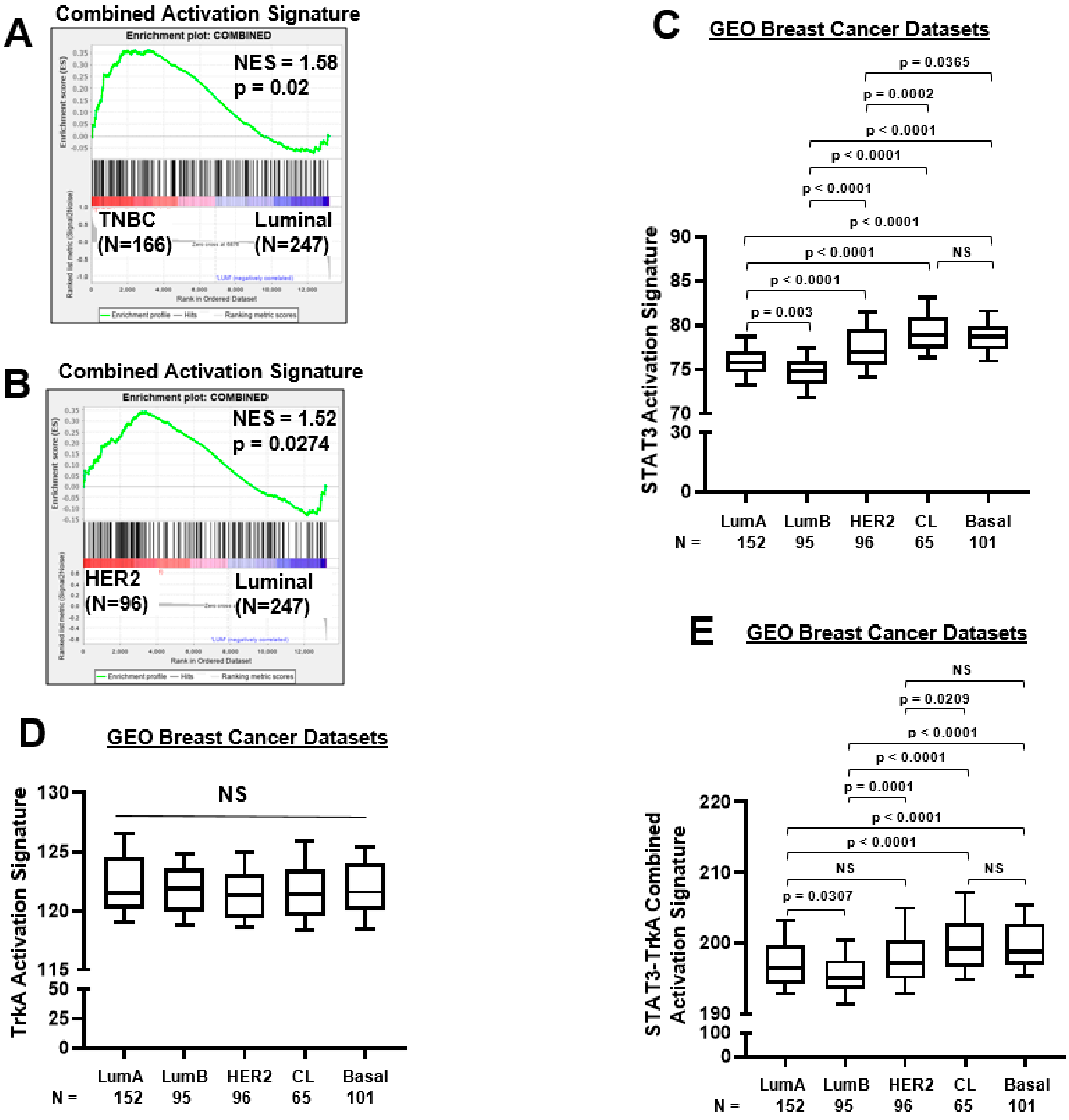

Figure S1. Expanded GSEA and datamining analyses of GEO breast cancer datasets using STAT3, TrkA, and combined STAT3-TrkA activation signatures. GSEA analyses of combined STAT3-TrkA signature in (**A**) triple-negative and (**B**) HER2-enriched breast cancers. (**C**) JAK2-STAT3 signature alone, (**D)** TrkA Activation Signature alone, or (**E**) combined STAT3-TrkA activation signature in breast cancer patients from GEO database, sorted into five breast cancer subtypes (Luminal A, Luminal B, HER2, Claudin-low, basal).

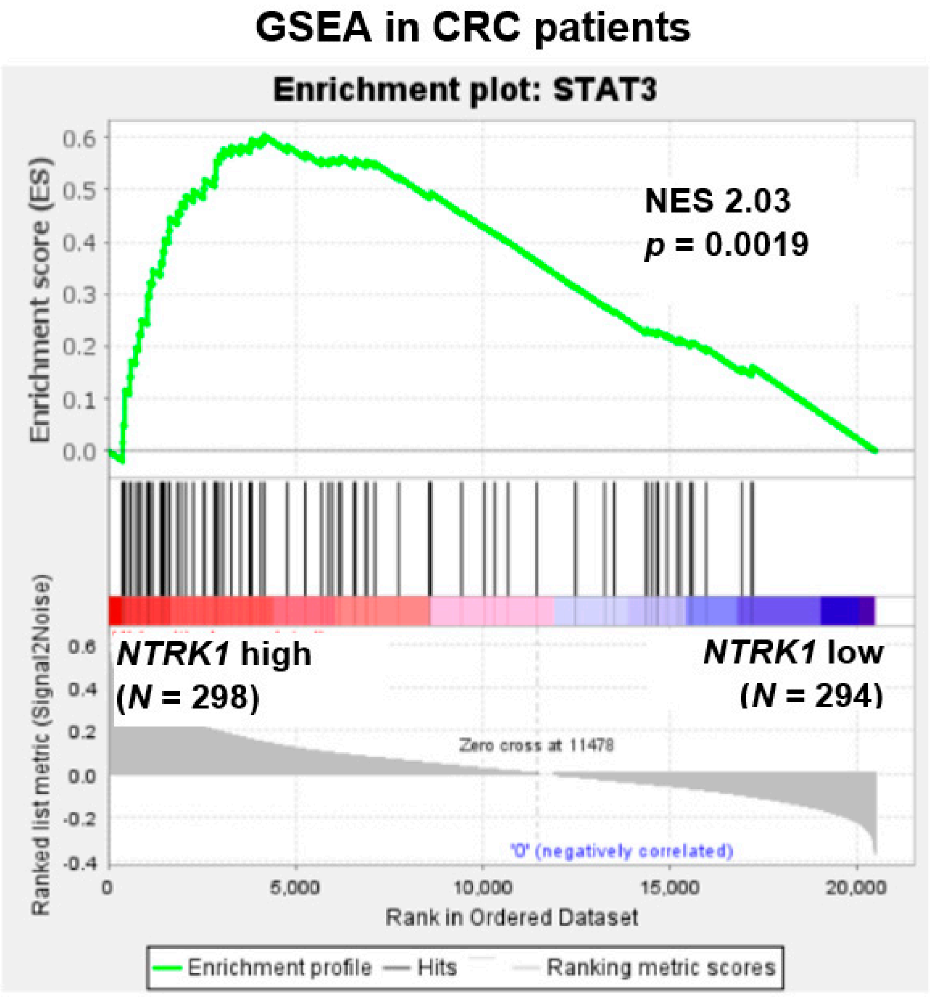

Figure S2. GSEA of STAT3 activation signature in colorectal cancer (CRC) patients. Patients were stratified based on median NTRK1 mRNA expression.

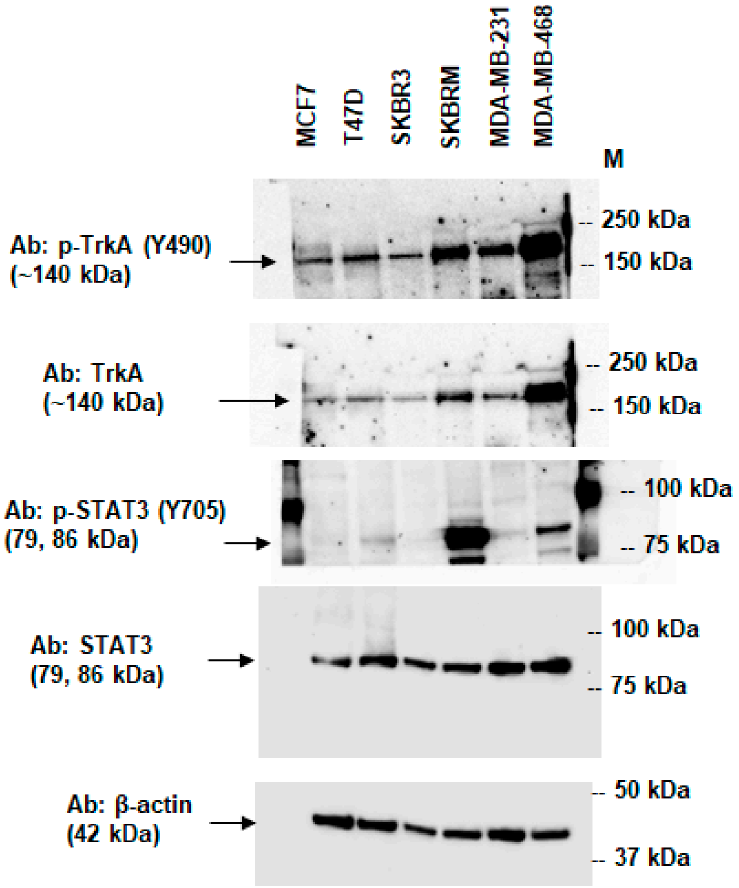

Figure S3. Original Western blot images for Figure 1C. (M = Protein standard marker).

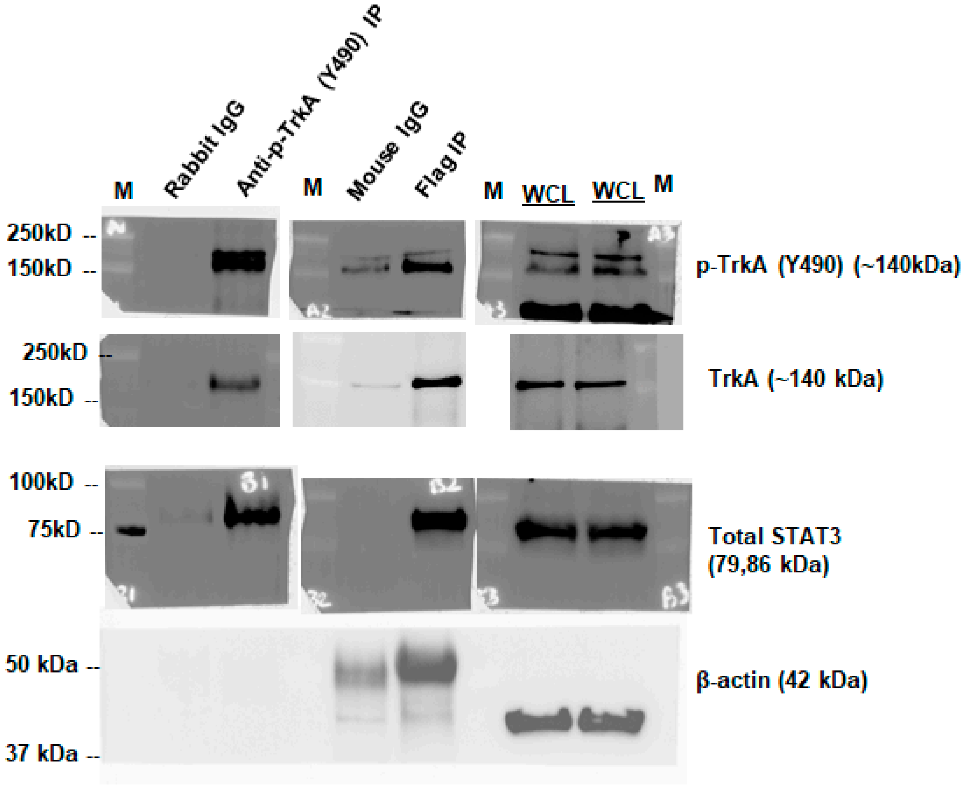

Figure S4. Original Western blot images for Figure 2A and 2B. (M = Protein Standard Marker).

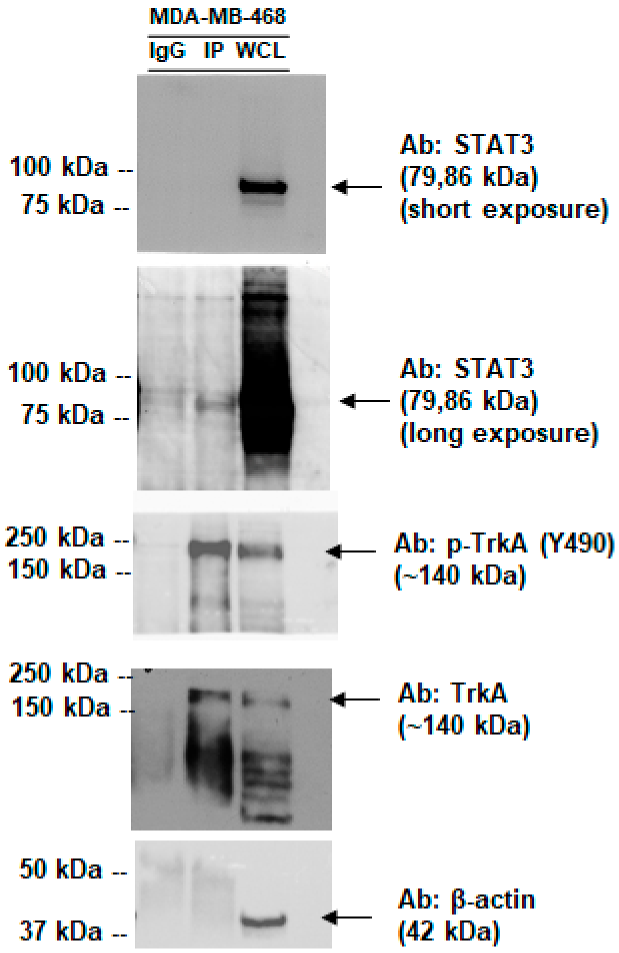

Figure S5. Original Western blot images for Figure 2C. (M = Protein standard marker).

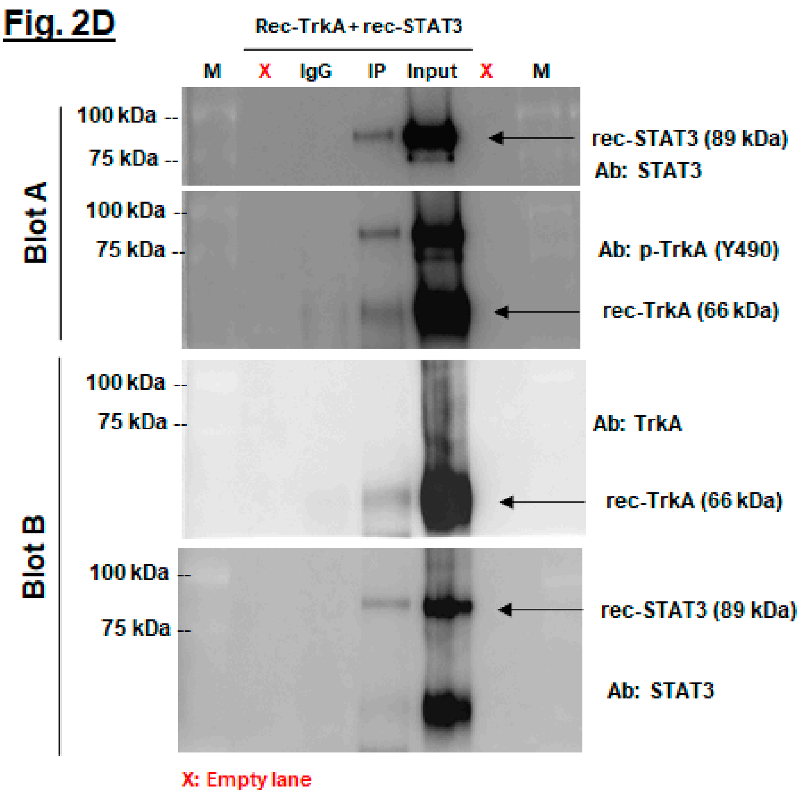

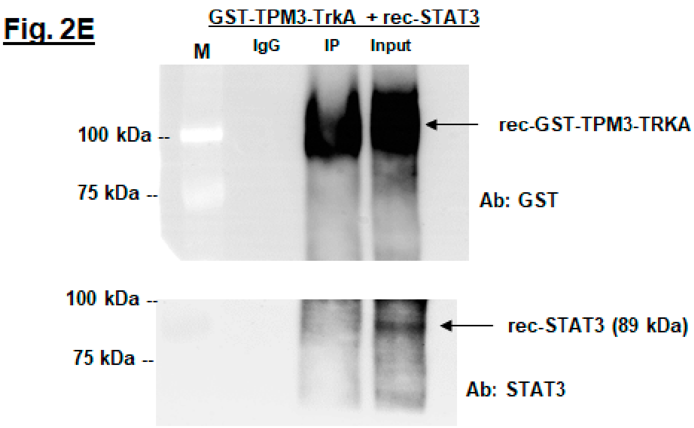

Figure S6. Original Western blot images for Figures 2D and 2E. (M = Protein standard marker).

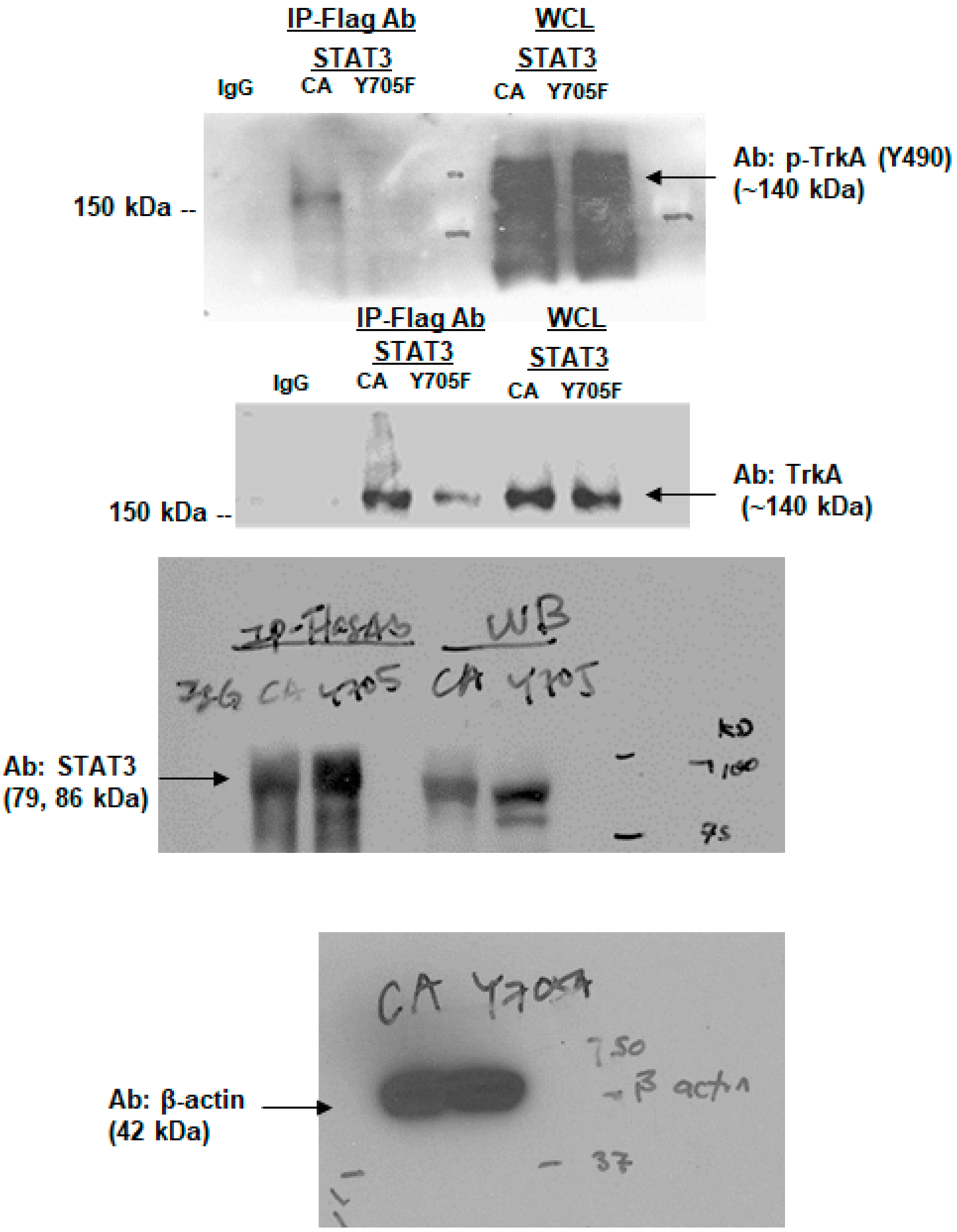

Figure S7. Original Western blot images for Figure 2F. (M = Protein standard marker).

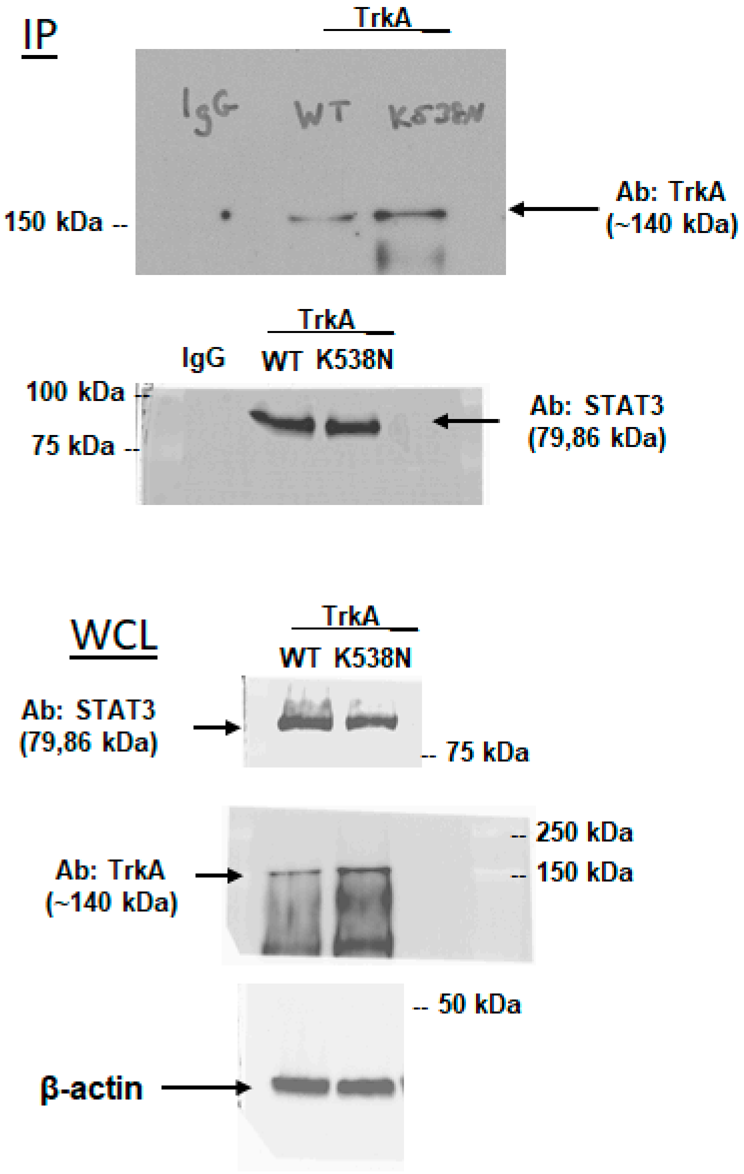

Figure S8. Original Western blot images for Figure 2G. (M = Protein standard marker).

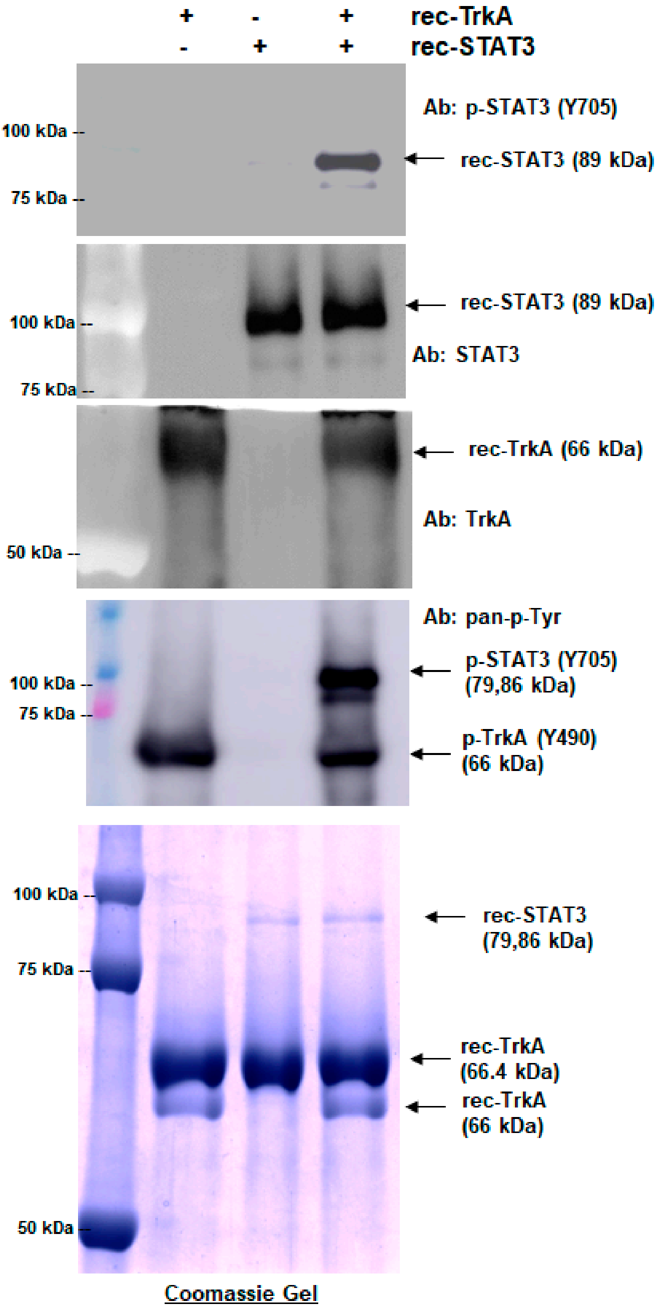

Figure S9. Original Western blot images for Figure 3A.

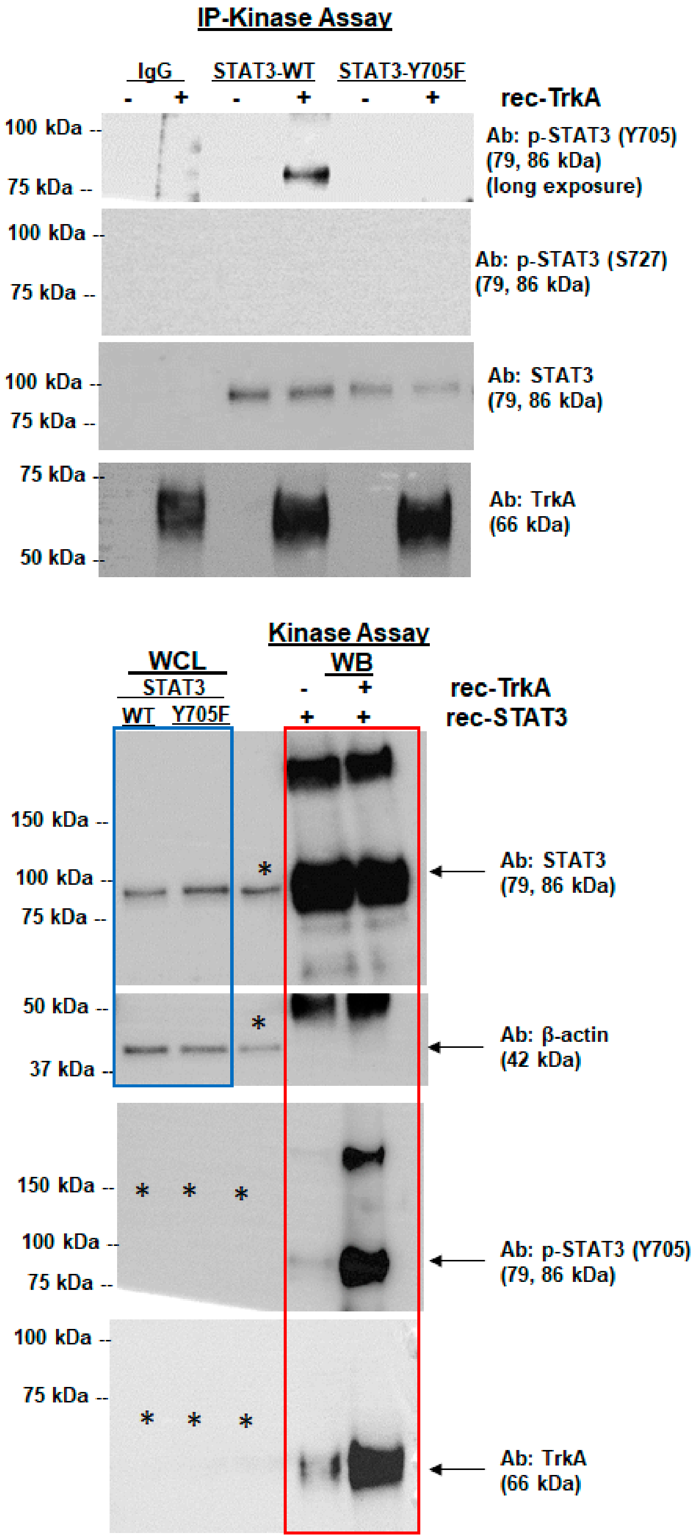

Figure S10. Original western blot images for Figure 3B. Portions of blots used for whole cell lysate (WCL; blue) and cell-free TrkA kinase assay western blot (red) have been highlighted. (*, irrelevant lanes).

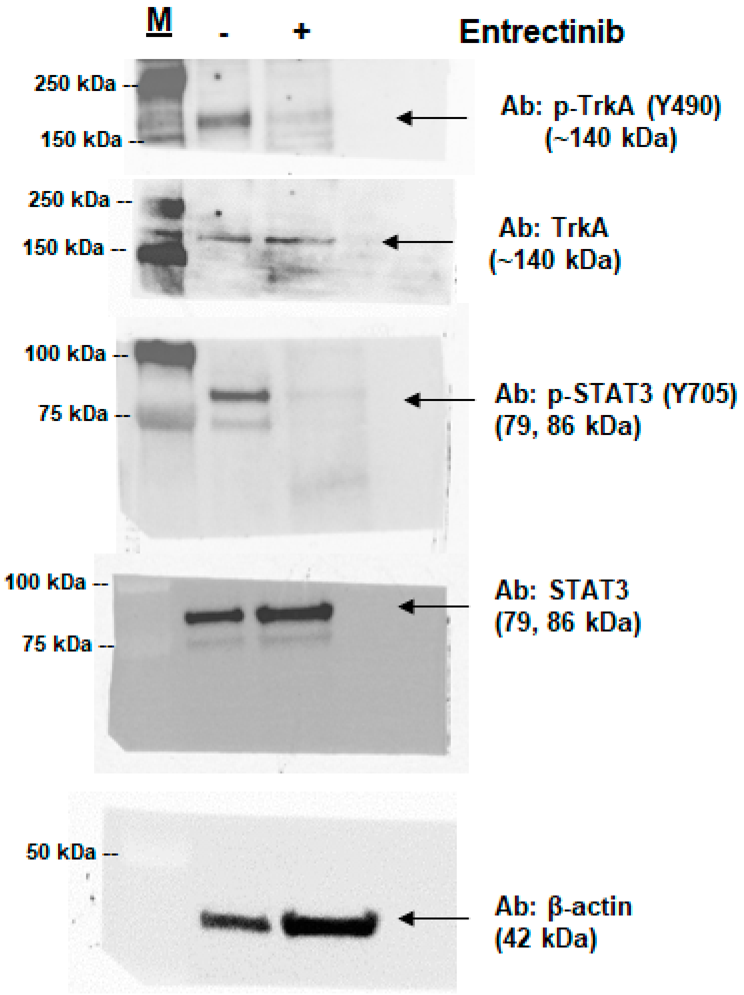

Figure S11. Original Western blot images for Figure 3C. (M = Protein standard marker).

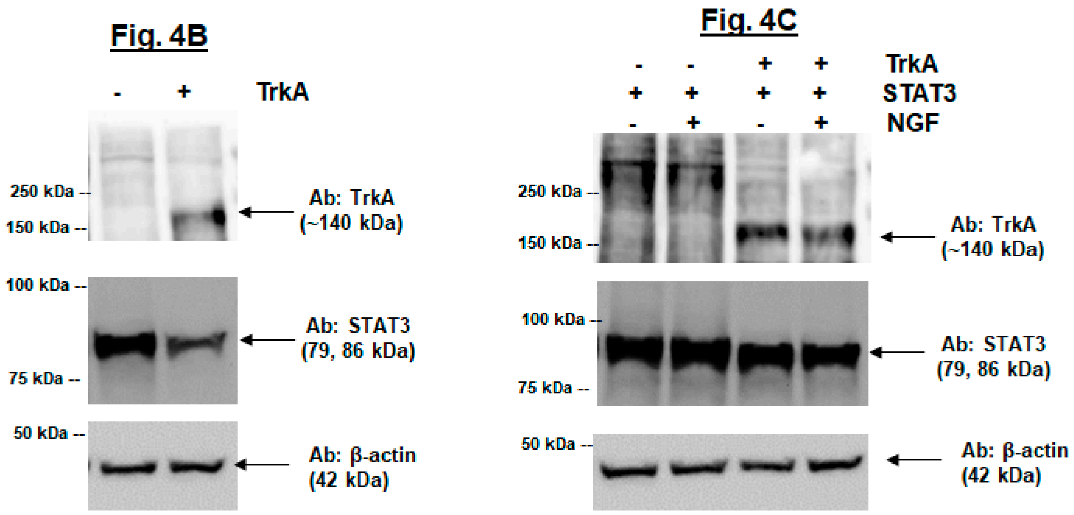

Figure S12. Original Western blot images for Figure 4B-4C. (M = Protein standard marker).

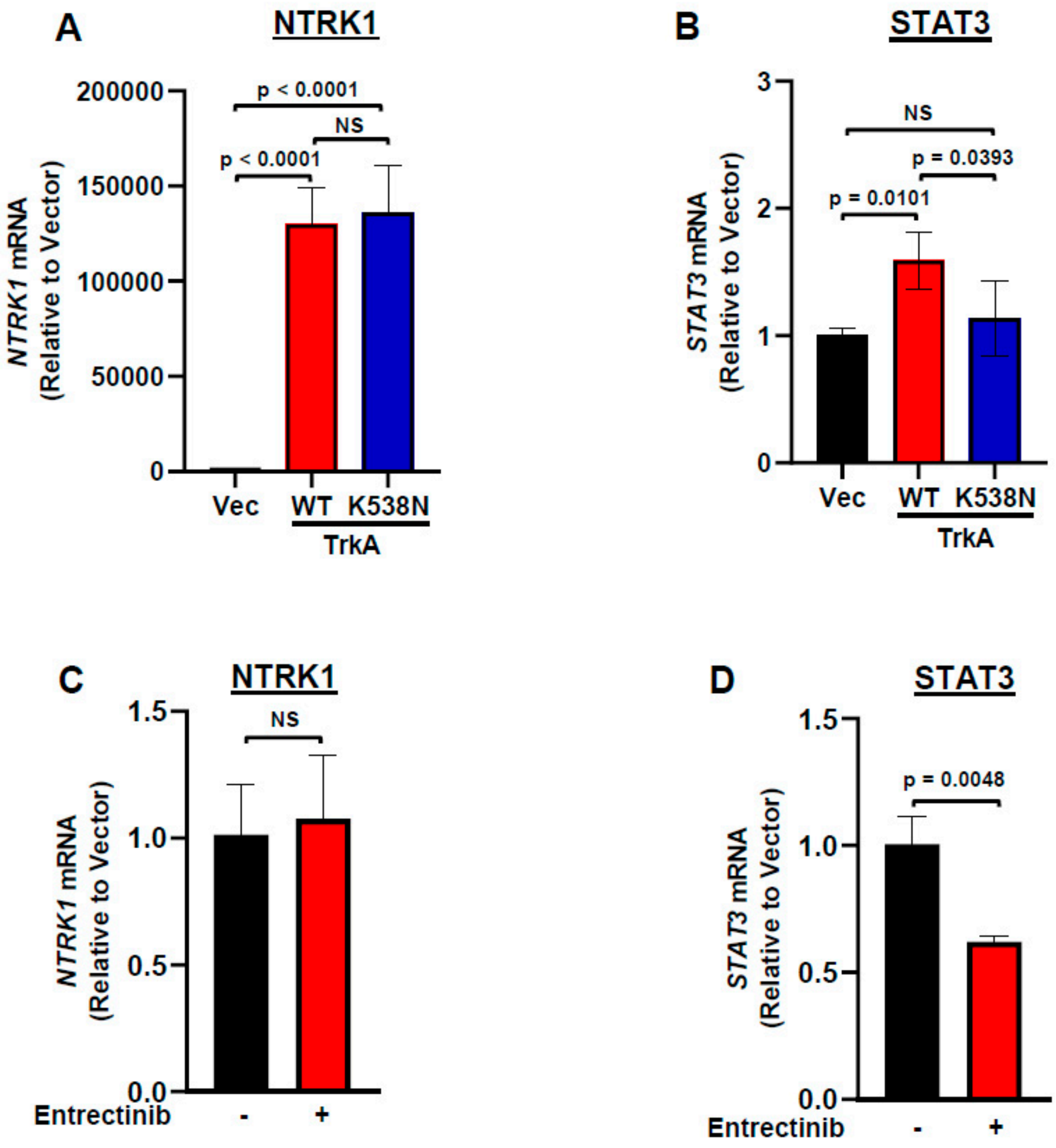

Figure S13. mRNA levels of NTRK1 and STAT3 genes upon overexpression or inhibition of TrkA.

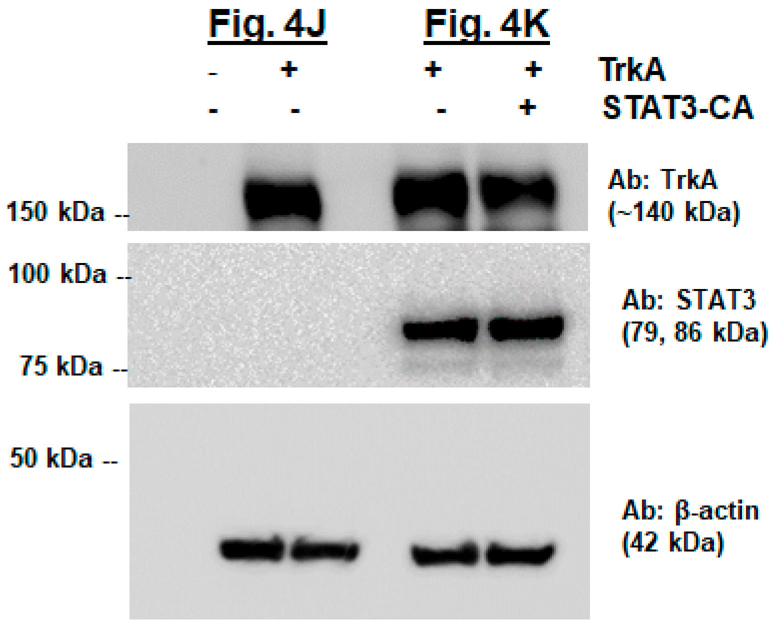

Figure S14. Original Western blot images for Figures 4J–4K.

Table S1. Site-directed mutagenesis primers.

Mutation	Primer Sequences (5′-3′)
STAT3-F705Y (For)	CAGGTAGCGCTGCCCCATACCTGAAGACCAAGTTTATC
STAT3-F705Y (Rev)	GATAAACTTGGTCTTCAGGTATGGGGCAGCGCTACCTG
TrkA-K538N (For)	GCTGGTGGCTGTCAATGCACTGAAGGAGG
TrkA-K538N (Rev)	CCTCCTTCAGTGCATTGACAGCCACCAGC

Table S2. Antibodies.

Antibodies			
**Target**	**Supplier and cat. No.**	**Species**	**Dilution**
Anti-p-TrkA (Y490)	Cell Signaling, #9141	Rabbit	1:1000 (WB); 1:25 (IF)
Anti-p-TrkA (Y490)	ThermoFisher, #PA5-104674	Rabbit	1:25 (IHC)
Anti-p-TrkA (Y490)	Sigma, #T9691	Rabbit	1:50 (IF)
Anti-p-STAT3 (Y705)	Cell Signaling, #9131	Rabbit	1:1000 (WB); 1:100 (IF)
Anti-p-STAT3 (Y705)	Cell Signaling, #4113	Mouse	1:1000 (WB); 1.100 (IF)
Anti-p-STAT3 (Y705)	Sigma Aldrich, #SAB4300033	Rabbit	1:25 (IHC)
Anti-p-STAT3 (S727)	Cell Signaling, #9134	Rabbit	1:1000 (WB)
Anti-β-actin	Cell Signaling, #3700	Mouse	1:1000 (WB)
Anti-phospho-Tyrosine	Millipore Sigma, #05-321	Mouse	1:1000 (WB)
Anti-GST	Cell Signaling, #2625	Rabbit	1:200 (IP)
Anti-GST	Cell Signaling, #2624	Mouse	1:1000 (WB)
Anti-mouse, Alexa 546	ThermoFisher, #A-11030	Goat	1:700 (IF)
Anti-rabbit, Cy5.5	Abcam, #ab6942	Goat	1.700 (IF)
Anti-mouse, Alexa 488	Invitrogen, #A-11001	Goat	1:700 (IF)
Anti-rabbit, Alexa 568	Invitrogen, #A-11011	Goat	1:700 (IF)
Anti-mouse	Cell Signaling, #7076	Goat	1:5,000 (WB)
Anti-rabbit	Cell Signaling, #7074	Goat	1:5,000 (WB)

Table S3. RT-PCR primer sequences.

RT-PCR primer sequences		
**Gene**	**Forward Primer (5′–3′)**	**Reverse Primer (5′–3′)**
Sox2	GGAGTTGTCAAGGCAGAGAAGAG	GAGAGAGGCAAACTGGAATC
myc	TGGTCTTCCCCTACCCTCTCAAC	GGCTCCTGGCAAAAGGTCAGAGTCTGGATC
GAPDH	ACTGCCAACGTGTCAGTGG	GTGTCGCTGTTGAAGTCAGA
